# Supplementary figures and images for: Combined model-free and model-sensitive reinforcement learning in non-human primates
Source: PLoS Comput Biol. 2020 Jun 22;16(6):e1007944. doi: 10.1371/journal.pcbi.1007944 (PMC7332075; doi:10.1371/journal.pcbi.1007944)

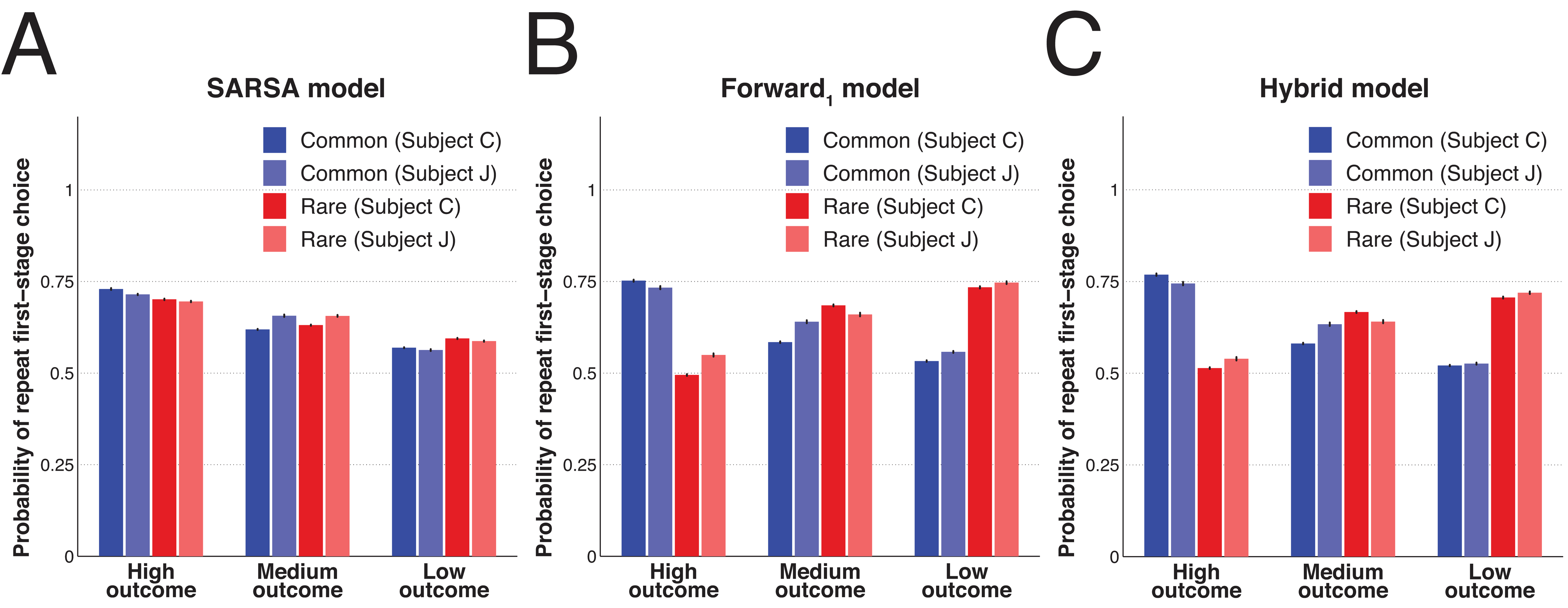

Supplement: S1 Fig — Simulated repetition probabilities as a function of outcome level and transition type for the best pure model-free SARSA model (A), the best pure model-sensitive Forward1 model (B) and the best Hybrid model (C). Values were averaged across all sessions, and across 100 simulation runs for each session using the parameters best fit to each subject’s data within each class of model (and respecting the exact same reward structure). Error bars depict SEM. (TIF) [file pcbi.1007944.s001.tif]

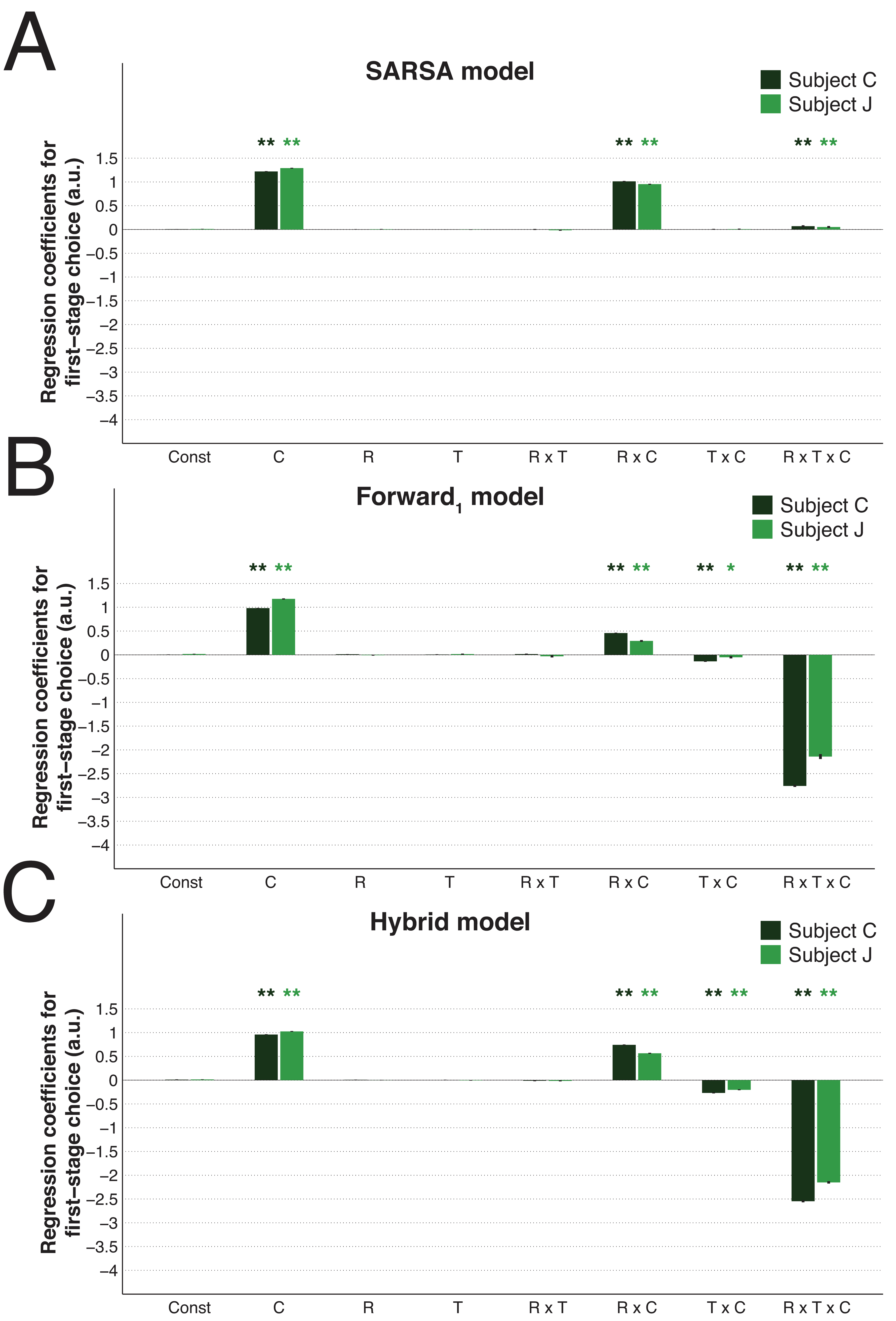

Supplement: S2 Fig — The predictors used were: Const (constant term) captured any potential first-stage picture bias; C (previous first-stage choice; 1 = car picture, 0 = watering can picture) modelled a potential independent tendency to stick with the same option from trial to trial; R (previous outcome level; assumed as continuous and with low = 1, medium = 2, high = 3), T (previous transition; rare = 1, common = 0) and R × T, measured any potential preference in first-stage picture choice given the previous outcome level, the previous transition and the interaction effect of both, respectively; R × C, T × C and R × T × C are the predictors of interest and quantify the main effects of reward, transition and the reward × transition interaction effect, respectively. All predictors were mean centred and continuous variables were also scaled by dividing them by two standard deviations (adjustments made before the computation of the interaction terms). Results for simulated choice behaviour (100 simulations per session for each subject and respecting the exact same reward structure) generated using the best-fitted mixed-effects parameters of the pure model-free SARSA model (A), pure model-sensitive Forward1 model (B) and Hybrid model (C). To note that the Hybrid model results are much closer to the MS-RL simulations as simulations used the parameters best fit to the subjects’ data and the MS weight estimated was close to 90%. Bar and error bar values correspond, respectively, to the mean and SE of the fixed-effects coefficients. ** for α = 0.01 and * for α = 0.05 in two-tailed one sample t-test with null-hypothesis mean equal to zero for the fixed-effects coefficients. (TIF) [file pcbi.1007944.s002.tif]

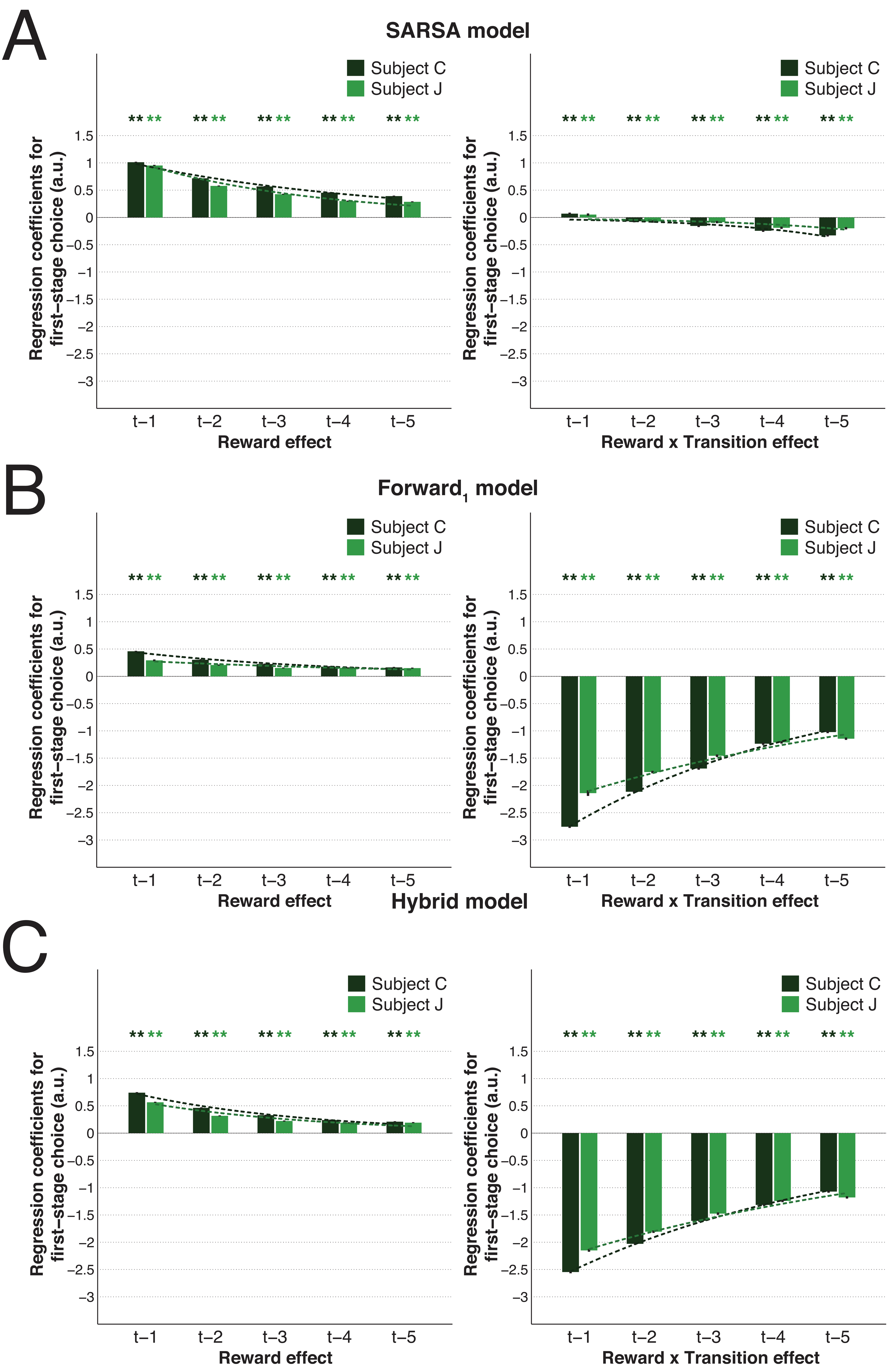

Supplement: S3 Fig — Multiple logistic regression results on first-stage simulated choice data (100 simulations per session for each subject and respecting the exact same reward structure) generated using the best-fitted mixed-effects parameters of the pure model-free SARSA model (A), pure model-sensitive Forward1 model (B) and Hybrid model (C) for the main effect of reward (left column) and reward × transition interaction term (right column) from the five previous trials. Bar and error bar values correspond, respectively, to the mean and SE of the fixed-effects coefficients. Dashed lines illustrate the exponential best fit on the mean fixed-effects coefficients of each trial into the past. ** for α = 0.01 and * for α = 0.05 in two-tailed one sample t-test with null-hypothesis mean equal to zero for the fixed-effects estimates. (TIF) [file pcbi.1007944.s003.tif]

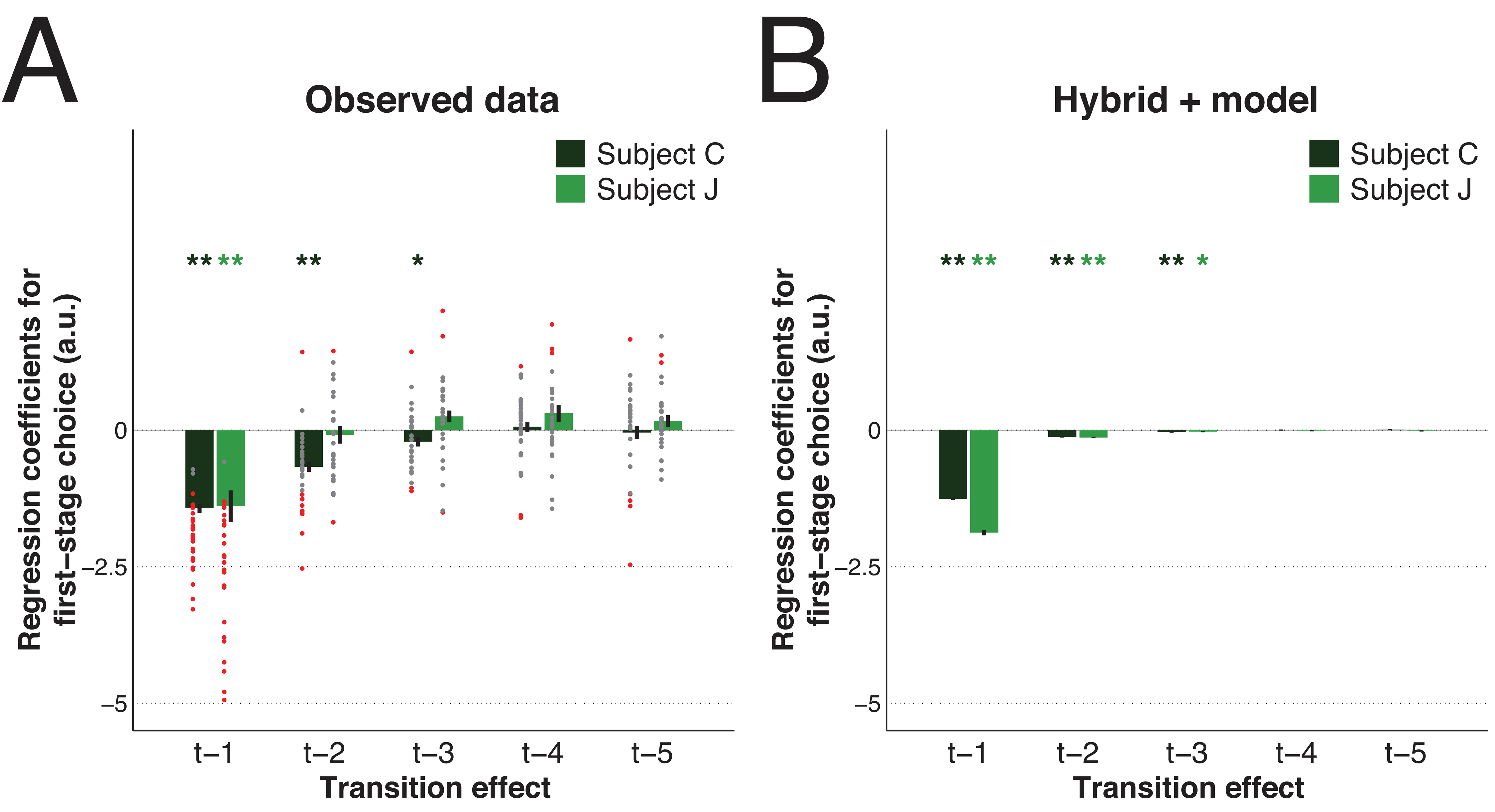

Supplement: S4 Fig — Results of the main effect of transition from the five previous trials obtained in the logistic regression on observed first-stage choice (A) and on first-stage simulated choice data (B) generated using the best-fitted mixed-effects parameters of the Hybrid+ model (100 simulations per session for each subject and respecting the exact same reward structure). Dots represent the fixed-effects coefficients for each session (coloured red when p < 0.05 and grey otherwise). Bar and error bar values correspond, respectively, to the mixed-effect coefficients and their SE. ** α = 0.01 and * α = 0.05 in two-tailed one sample t-test with null-hypothesis mean equal to zero for the fixed-effects coefficients. (TIF) [file pcbi.1007944.s004.tif]

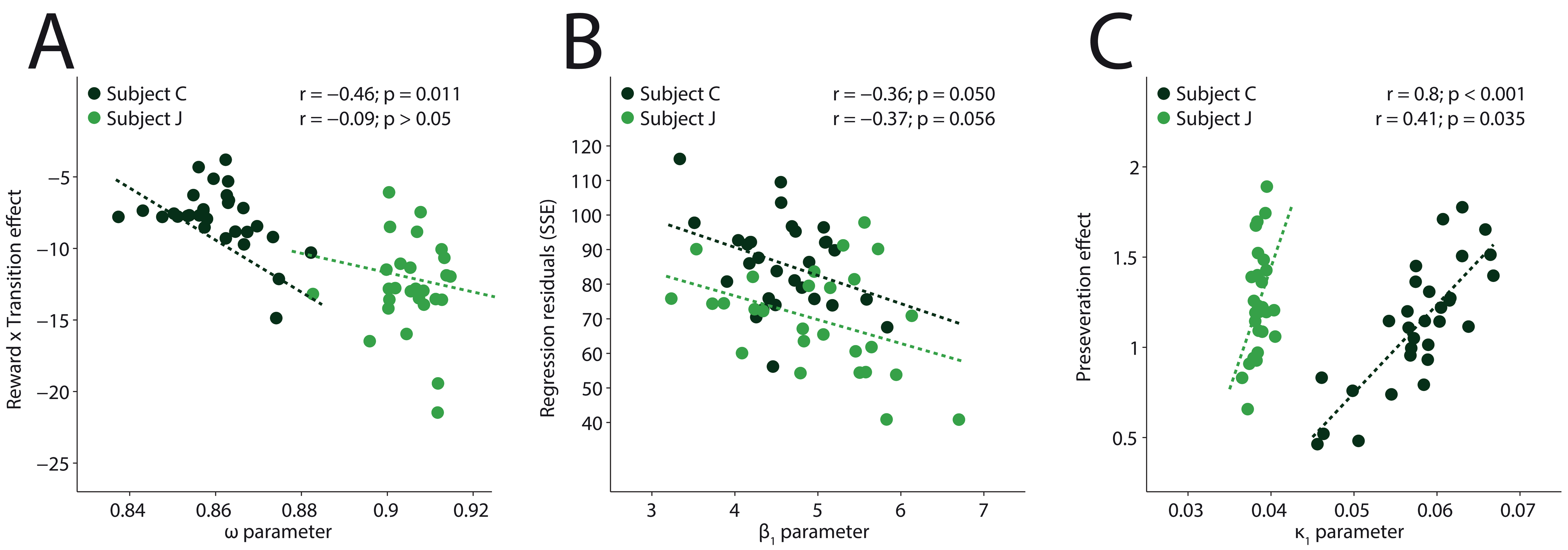

Supplement: S5 Fig — (A) The greater the model-sensitive weight parameter ω obtained from the Hybrid+ model fitting, the more negative (i.e. the stronger the effect in the logistic regression) the regression coefficient for the reward × transition interaction. (B) Relationship between the inverse temperature parameter at first-stage choice β1 obtained from the Hybrid+ model fitting and the residual values from the regression model (the greater the β1 parameter, the better the logistic regression fit). (C) Positive correlation between the computational preseveration κ1 parameter and the regression coefficient for repeat first-stage choice independently of reward and transition. Dashed lines represent the regression line of the fit for each individual subject. r is the Pearson’s linear correlation coefficients and p is the p-values: top values are for subject C and bottom values are for subject J. (TIF) [file pcbi.1007944.s005.tif]

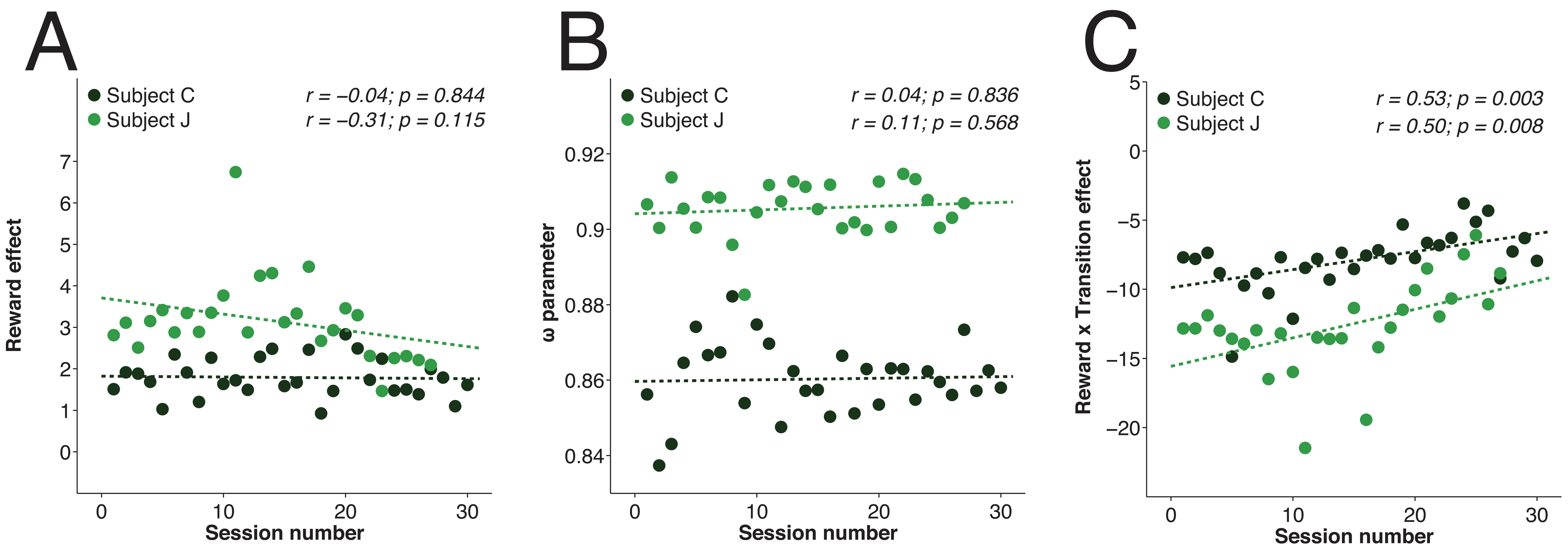

Supplement: S6 Fig — Across time and for both subjects, no significant decrease in the regression coefficients for the reward effect (A) or model-sensitive weight parameter ω (B) was found (both simulated results also with p > 0.05). However, a significant reduction was found for the effect of the regression coefficients for the reward × transition effect (C) with time (note that the more positive the regression coefficient the weaker the effect; simulated results: r = −0.01/−0.24, p = 0.959/0.228 for C/J). Dashed lines represent the regression line of the fit for each individual subject. r is the Pearson’s linear correlation coefficients and p is the p-values; top values are for subject C and bottom values are for subject J. (TIF) [file pcbi.1007944.s006.tif]

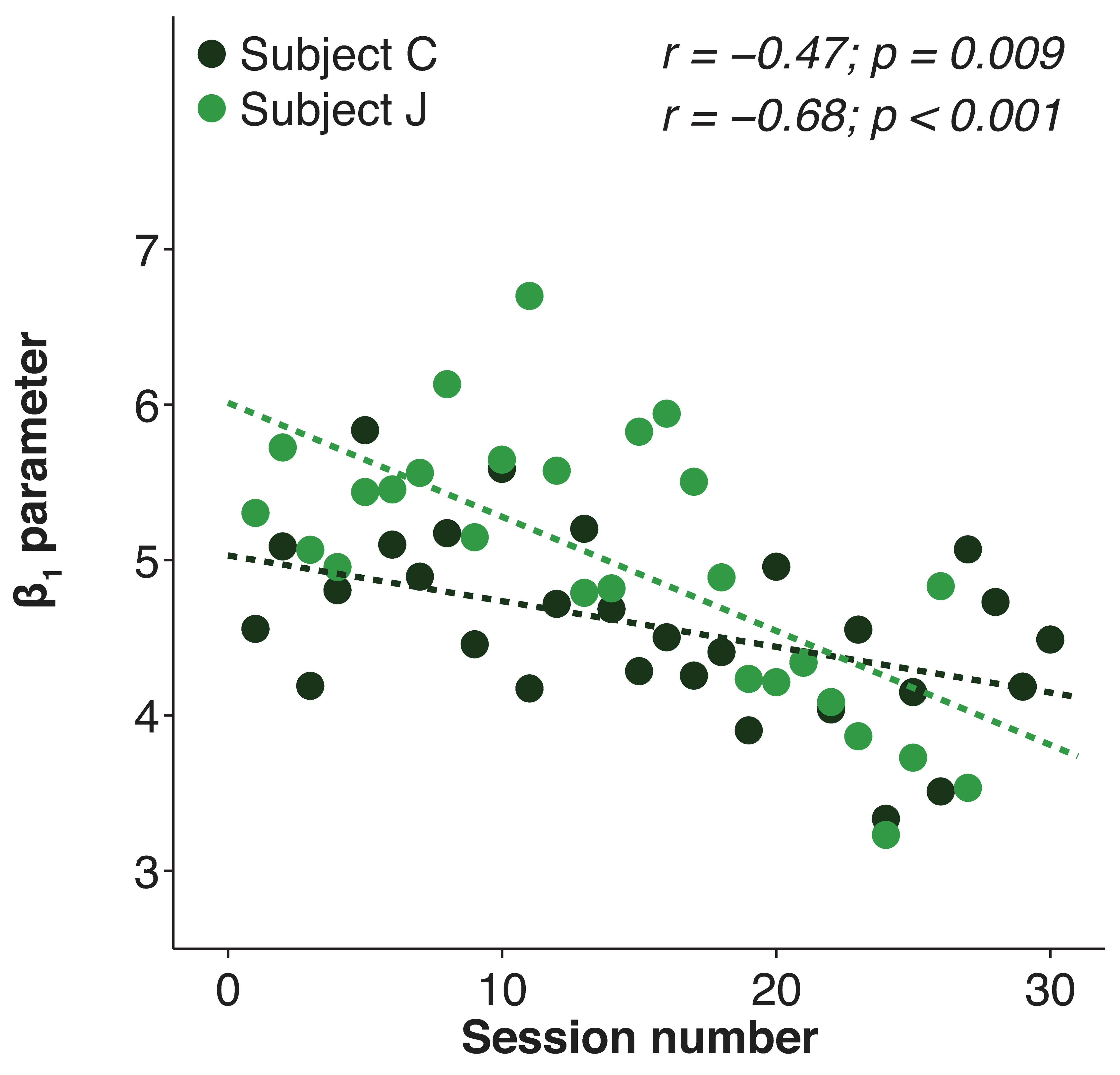

Supplement: S7 Fig — As the number of sessions performed increased, subjects got progressively more stochastic (smaller inverse temperature values in observed behaviour; simulated results did not present such decrement: r = −0.02/−0.06, p = 0.898/0.768) in their choice behaviour. Dashed lines represent the regression line of the fit for each individual subject. r is the Pearson’s linear correlation coefficients and p is the p-values; top values are for subject C and bottom values are for subject J. (TIF) [file pcbi.1007944.s007.tif]

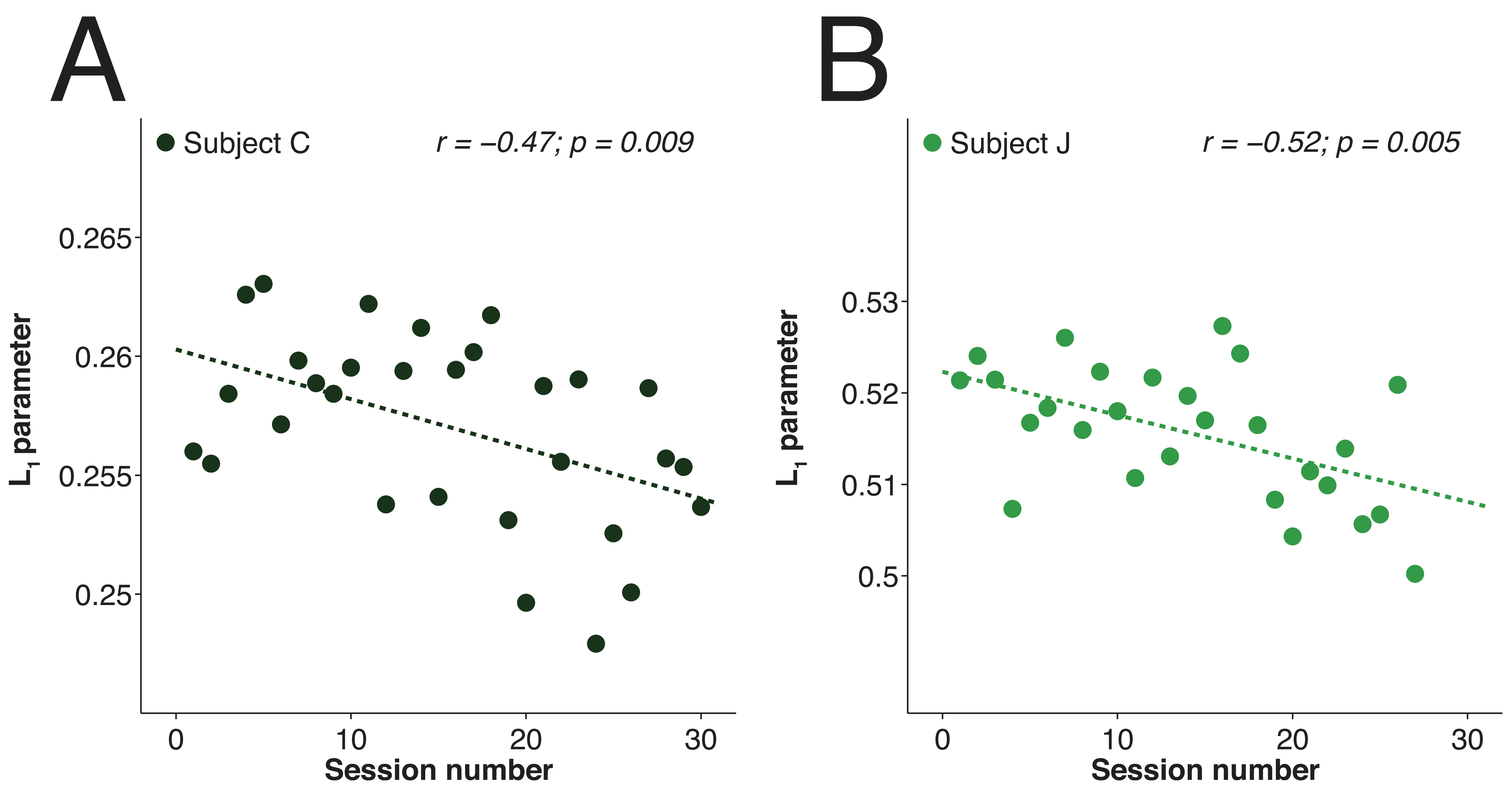

Supplement: S8 Fig — As the number of sessions performed increased, the L1 parameter value got progressively smaller (i.e., less strength of the reinforcement by previous trial’s high reward) in both subject C (A) and subject J (B). Dashed lines represent the regression line of the fit for each individual subject. r is the Pearson’s linear correlation coefficients and p is the p-values. (TIF) [file pcbi.1007944.s008.tif]

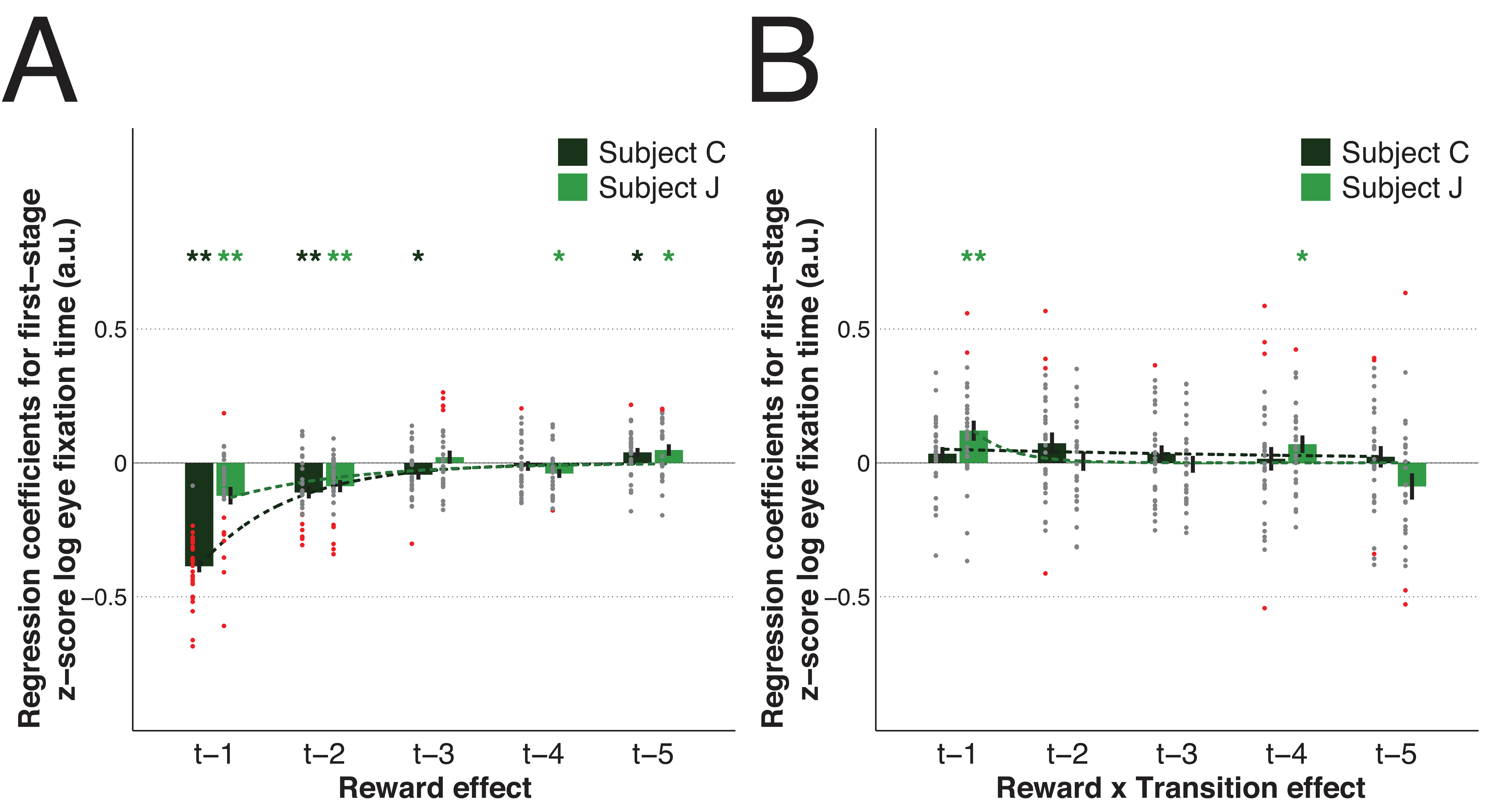

Supplement: S9 Fig — Multiple linear regression results on z-scores of log transformed first-stage eye fixation time (high z-scores indicate slow first eye fixation attempt) with the contributions of the reward main effect (A) and reward × transition interaction term (B) from the five previous trials. Dots represent the fixed-effects coefficients for each session (coloured red when p < 0.05 and grey otherwise). Bar and error bar values correspond, respectively, to the mean value of the fixed-effect coefficients and its SEM. Dashed lines illustrate the exponential best fit on the mean fixed-effects coefficients of each trial into the past. ** for α = 0.01 and * for α = 0.05 in two-tailed one sample t-test with null-hypothesis mean equal to zero for the fixed-effects coefficients. (TIF) [file pcbi.1007944.s009.tif]
