# Supplementary material for: Combined model-free and model-sensitive reinforcement learning in non-human primates
Source: PLoS Comput Biol. 2020 Jun 22;16(6):e1007944. doi: 10.1371/journal.pcbi.1007944 (PMC7332075; doi:10.1371/journal.pcbi.1007944)
Supplement: S1 Table — (PDF) [file pcbi.1007944.s010.pdf]

Predictors‡	Fixed-effects* C	Fixed-effects* J	Mixed-effects† C	Mixed-effects† J
Const	-0.11 (0.06)	-0.08 (0.08)	-0.06 (0.05)	-0.07 (0.08)
C_{t-1}	0.71 (0.06)	0.91 (0.07)§	0.56 (0.05)§	0.57 (0.07)§
R_{t-1}	-0.35 (0.06)§	-0.32 (0.11)§	-0.22 (0.06)§	-0.12 (0.22)
T_{t-1}	0.07 (0.05)	0.07 (0.08)	0.01 (0.05)	0.05 (0.09)
$\mathrm{R}_{t-1} \times \mathrm{T}_{t-1}$	0.27 (0.12)	-0.18 (0.18)	0.21 (0.11)	-0.45 (0.18)¶
$R_{t-1} \times C_{t-1}$	*1.54 (0.10)*§	*3.26 (0.22)*§	*1.36 (0.09)*§	*2.82 (0.26)*§
$T_{t-1} \times C_{t-1}$	*-1.92 (0.11)*§	*-2.53 (0.21)*§	*-1.43 (0.08)*§	*-1.39 (0.29)*§
$R_{t-1} \times T_{t-1} \times C_{t-1}$	*-7.85 (0.50)*§	*-13.76 (0.70)*§	*-7.06 (0.39)*§	*-16.37 (1.22)*§
C_{t-2}	0.37 (0.05)§	0.17 (0.08)	0.32 (0.04)§	0.11 (0.07)
R_{t-2}	-0.05 (0.05)	0.03 (0.08)	-0.03 (0.05)	0.03 (0.08)
T_{t-2}	0.11 (0.06)	0.07 (0.07)	0.06 (0.04)	0.08 (0.05)
$\mathrm{R}_{t-2} \times \mathrm{T}_{t-2}$	0.28 (0.11)¶	0.17 (0.14)	0.14 (0.09)	0.14 (0.14)
$R_{t-2} \times C_{t-2}$	*0.70 (0.10)*§	*0.57 (0.12)*§	*0.62 (0.08)*§	*0.63 (0.13)*§
$T_{t-2} \times C_{t-2}$	*-0.72 (0.13)*§	*-0.13 (0.15)*	*-0.67 (0.09)*§	*-0.09 (0.16)*
$R_{t-2} \times T_{t-2} \times C_{t-2}$	*-2.75 (0.31)*§	*-2.68 (0.33)*§	*-2.43 (0.26)*§	*-2.11 (0.32)*§
C_{t-3}	0.17 (0.06)§	0.07 (0.09)	0.17 (0.05)§	0.06 (0.08)
R_{t-3}	0.12 (0.06)	-0.02 (0.08)	0.07 (0.04)	-0.03 (0.08)
T_{t-3}	0.15 (0.06)¶	0.07 (0.06)	0.12 (0.04)§	0.09 (0.05)
$\mathrm{R}_{t-3} \times \mathrm{T}_{t-3}$	0.09 (0.11)	-0.19 (0.15)	0.11 (0.10)	-0.14 (0.15)
$R_{t-3} \times C_{t-3}$	*0.31 (0.11)*§	*0.30 (0.17)*	*0.33 (0.08)*§	*0.28 (0.13)*¶
$T_{t-3} \times C_{t-3}$	*-0.26 (0.11)*¶	*0.19 (0.15)*	*-0.21 (0.09)*¶	*0.25 (0.11)*¶
$R_{t-3} \times T_{t-3} \times C_{t-3}$	*-1.33 (0.22)*§	*-1.31 (0.40)*§	*-1.26 (0.19)*§	*-1.48 (0.32)*§
C_{t-4}	0.05 (0.06)	-0.07 (0.07)	0.04 (0.04)	-0.05 (0.06)
R_{t-4}	0.03 (0.06)	-0.04 (0.06)	-0.19 (0.06)	-0.04 (0.05)
T_{t-4}	0.02 (0.06)	0.04 (0.06)	0.02 (0.06)	0.03 (0.05)
$\mathrm{R}_{t-4} \times \mathrm{T}_{t-4}$	0.04 (0.10)	-0.11 (0.15)	0.06 (0.09)	-0.17 (0.17)
$R_{t-4} \times C_{t-4}$	*0.23 (0.10)*¶	*0.07 (0.11)*	*0.20 (0.08)*¶	*0.17 (0.11)*
$T_{t-4} \times C_{t-4}$	*0.06 (0.12)*	*0.23 (0.14)*	*0.06 (0.09)*	*0.31 (0.15)*¶
$R_{t-4} \times T_{t-4} \times C_{t-4}$	*-0.70 (0.25)*§	*-0.89 (0.29)*§	*-0.66 (0.19)*§	*-1.02 (0.32)*§
C_{t-5}	0.06 (0.05)	0.15 (0.05)¶	0.06 (0.04)	0.11 (0.05)¶
R_{t-5}	0.10 (0.05)	-0.02 (0.05)	0.11 (0.04)§	-0.04 (0.06)
T_{t-5}	0.07 (0.05)	0.01 (0.05)	0.02 (0.04)	0.02 (0.05)
$\mathrm{R}_{t-5} \times \mathrm{T}_{t-5}$	0.05 (0.10)	-0.05 (0.16)	0.08 (0.09)	0.12 (0.15)
$R_{t-5} \times C_{t-5}$	*0.01 (0.12)*	*0.10 (0.10)*	*-0.01 (0.11)*	*0.01 (0.10)*
$T_{t-5} \times C_{t-5}$	*-0.02 (0.15)*	*0.22 (0.11)*	*-0.04 (0.12)*	*0.17 (0.11)*
$R_{t-5} \times T_{t-5} \times C_{t-5}$	*-0.50 (0.24)*¶	*0.22 (0.26)*	*-0.35 (0.22)*	*-0.34 (0.29)*

*Fixed-effects results are mean (SEM) of the regression coefficients across sessions. †Mixed-effects results are the regression coefficients (SE). ‡For the given trial t, the predictors used were: Const (constant term) captured any potential first-stage picture bias; C (previous first-stage choice; 1=car picture, 0=watering can picture) modelled a potential independent tendency to stick with the same option from trial to trial; R (previous outcome level; assumed as continuous and with low=1, medium=2, high=3), T (previous transition; rare=1, common=0) and R × T, measured any potential preference in first-stage picture choice given the previous outcome level, the previous transition and the interaction effect of both, respectively; R × C, T × C and R × T × C are the predictors of interest (in italic) and quantify the main effects of reward, transition and the reward × transition interaction effect, respectively. All predictors were mean centred and continuous variables were also scaled by dividing them by two SD (adjustments made before the computation of the interaction terms). §Significance at the 0.01 level. ¶Significance at the 0.05 level.
