# Supplementary material for: Combined model-free and model-sensitive reinforcement learning in non-human primates
Source: PLoS Comput Biol. 2020 Jun 22;16(6):e1007944. doi: 10.1371/journal.pcbi.1007944 (PMC7332075; doi:10.1371/journal.pcbi.1007944)
Supplement: S2 Table — (PDF) [file pcbi.1007944.s011.pdf]

	Fixed-effects BIC sum		Mixed-effects BIC_{int}	
Parameters*	C	J	C	J
α, β, κ_1	35873	34670	35717	34538
α, β, κ_2	36581	35191	36444	35170
α, β, κ	35814	34133	35679	34043
α, β, λ	36330	35157	36149	35171
α_1, α_2, β	36511	35671	36494	35700
α, β_1, β_2	36532	35682	36393	35596
α_1, α_2, β_1, β_2	36615	35770	36464	35775
α_1, α_2, β, κ_1	35822	34759	35689	34588
α_1, α_2, β, κ_2	36584	35295	36430	35274
α_1, α_2, β, κ	35821	34252	35687	34087
α_1, α_2, β, λ	36335	35267	36116	35231
α, β_1, β_2, κ_1	35785	34742	35455	34458
α, β_1, β_2, κ_2	36619	35306	36348	35175
α, β_1, β_2, κ	35934	34267	35637	34022
α, β_1, β_2, λ	36447	35260	36163	35176
α, β, κ_1, κ_2	35908	34256	35624	34069
α, β, κ_1, λ	35723	34415	35424	34185
α, β, κ_2, λ	36403	34825	36095	34791
α, β, κ, λ	**35697**†	**33931**†	35420	**33750**†
α_1, α_2, β_1, β_2, κ_1	35895	34859	35494	34509
α_1, α_2, β_1, β_2, κ_2	37797	35690	36765	35698
α_1, α_2, β_1, β_2, κ	35949	34388	35723	34088
α_1, α_2, β_1, β_2, λ	36478	35360	36142	35272
α, β_1, β_2, κ_1, κ_2	35873	34370	35408	34049
α, β_1, β_2, κ_1, λ	35794	34553	35319	34183
α, β_1, β_2, κ_2, λ	36531	34888	36117	34774
α, β_1, β_2, κ, λ	35856	34058	35385	33754
α_1, α_2, β, κ_1, κ_2	35891	34373	36074	34133
α_1, α_2, β, κ_1, λ	35707	34539	35356	34240
α_1, α_2, β, κ_2, λ	36426	34936	36074	34856
α_1, α_2, β, κ, λ	35725	34061	35382	33788
α, β, κ_1, κ_2, λ	35784	34055	35348	33780
α_1, α_2, β_1, β_2, κ_1, κ_2	35983	34494	35439	34087
α_1, α_2, β_1, β_2, κ_1, λ	35858	34677	35311	34233
α_1, α_2, β_1, β_2, κ_2, λ	36566	34994	36090	34865
α_1, α_2, β_1, β_2, κ, λ	35858	34188	35331	33796
α, β_1, β_2, κ_1, κ_2, λ	35880	34186	35266	33766
α_1, α_2, β, κ_1, κ_2, λ	35791	34186	35287	33824
α_1, α_2, β_1, β_2, κ_1, κ_2, λ	35946	34312	**35260**†	33825

*Abbreviations: learning rate for first-stage (α_1) and second-stage (α_2); α is when $\alpha_1 = \alpha_2$; inverse temperature for first-stage (β_1) and second-stage (β_2); β is when $\beta_1 = \beta_2$; perseveration for first-stage (κ_1) and second-stage (κ_2); κ is when $\kappa_1 = \kappa_2$; eligibility trace (λ).

†Best fitting $SARSA$ model variant for the respective subject and analysis type.
