# Supplementary material for: Combined model-free and model-sensitive reinforcement learning in non-human primates
Source: PLoS Comput Biol. 2020 Jun 22;16(6):e1007944. doi: 10.1371/journal.pcbi.1007944 (PMC7332075; doi:10.1371/journal.pcbi.1007944)
Supplement: S3 Table — (PDF) [file pcbi.1007944.s012.pdf]

Parameters *	Fixed-effects BIC sum		Mixed-effects BIC_{int}	
	C	J	C	J
α, β	36445	35620	36415	35706
α, β, κ_1	35767	34668	35633	34574
α, β, κ_2	36508	35252	36354	35283
α, β, κ	35736	34162	35620	34101
α, β, λ	36357	35399	36245	35525
α_1, α_2, β	36484	35729	36428	35749
α, β_1, β_2	36501	35731	36408	35764
α_1, α_2, β_1, β_2	36609	35836	36431	35878
α_1, α_2, β, κ_1	35778	34788	35614	34607
α_1, α_2, β, κ_2	36566	35372	36373	35427
α_1, α_2, β, κ	35792	34297	35615	34088
α_1, α_2, β, λ	36420	35502	36265	35556
α, β_1, β_2, κ_1	35761	34780	35483	34589
α, β_1, β_2, κ_2	36587	35357	36370	35383
α, β_1, β_2, κ	35878	34300	35609	34084
α, β_1, β_2, λ	36458	35494	36367	35528
α, β, κ_1, κ_2	35819	34281	35560	34127
α, β, κ_1, λ	35721	34571	35486	34470
α, β, κ_2, λ	36434	35068	36206	35160
α, β, κ, λ	**35709**†	**34095**†	35502	33942
α_1, α_2, β_1, β_2, κ_1	35891	34909	35559	34545
α_1, α_2, β_1, β_2, κ_2	37797	35773	36742	35552
α_1, α_2, β_1, β_2, κ	35925	34426	35829	34119
α_1, α_2, β_1, β_2, λ	36543	35590	36312	35612
α, β_1, β_2, κ_1, κ_2	35849	34409	35451	34140
α, β_1, β_2, κ_1, λ	35799	34713	35411	34352
α, β_1, β_2, κ_2, λ	36540	35121	36301	35121
α, β_1, β_2, κ, λ	35856	34220	35467	**33935**†
α_1, α_2, β, κ_1, κ_2	35856	34413	35537	34169
α_1, α_2, β, κ_1, λ	35762	34704	35445	34423
α_1, α_2, β, κ_2, λ	36510	35143	36227	35169
α_1, α_2, β, κ, λ	35786	34224	35492	33969
α, β, κ_1, κ_2, λ	35788	34217	35437	34006
α_1, α_2, β_1, β_2, κ_1, κ_2	35979	34546	36026	34133
α_1, α_2, β_1, β_2, κ_1, λ	35904	34844	35399	34432
α_1, α_2, β_1, β_2, κ_2, λ	36566	34994	36084	34888
α_1, α_2, β_1, β_2, κ, λ	35907	34350	35524	33990
α, β_1, β_2, κ_1, κ_2, λ	35884	34347	35429	34010
α_1, α_2, β, κ_1, κ_2, λ	35847	34346	35404	34038
α_1, α_2, β_1, β_2, κ_1, κ_2, λ	35992	34473	**35376**†	34008

*Abbreviations: learning rate for first-stage (α_1) and second-stage (α_2); α is when $\alpha_1 = \alpha_2$; inverse temperature for first-stage (β_1) and second-stage (β_2); β is when $\beta_1 = \beta_2$; perseveration for first-stage (κ_1) and second-stage (κ_2); κ is when $\kappa_1 = \kappa_2$; eligibility trace (λ).

†Best fitting Q-learning model variant for the respective subject and analysis type.
