# Supplementary material for: Combined model-free and model-sensitive reinforcement learning in non-human primates
Source: PLoS Comput Biol. 2020 Jun 22;16(6):e1007944. doi: 10.1371/journal.pcbi.1007944 (PMC7332075; doi:10.1371/journal.pcbi.1007944)
Supplement: S4 Table — (PDF) [file pcbi.1007944.s013.pdf]

| Model* | Parameters† | Fixed-effects $BIC$ sum | | Mixed-effects $BIC_{int}$ | |
|---|---|---|---|---|---|
| | | C | J | C | J |
| $Forward_1$ | | | | | |
| | $\alpha_2$, $\beta$ | 35298 | 34824 | 35275 | 34868 |
| | $\alpha_2$, $\beta_1$, $\beta_2$ | 34630 | 33708 | 34548 | 33743 |
| | $\alpha_2$, $\beta$, $\kappa_1$ | 34737 | 34038 | 34610 | 33965 |
| | $\alpha_2$, $\beta$, $\kappa_2$ | 35425 | 34572 | 35271 | 34619 |
| | $\alpha_2$, $\beta$, $\kappa$ | 34818 | 33634 | 34701 | 33616 |
| | $\alpha_2$, $\beta$, $\kappa_1$, $\kappa_2$ | 34856 | 33753 | 34595 | 33658 |
| | $\alpha_2$, $\beta_1$, $\beta_2$, $\kappa_1$ | 34418 | 33437 | 34176 | 33345 |
| | $\alpha_2$, $\beta_1$, $\beta_2$, $\kappa_2$ | 34715 | 33342 | 34499 | 33309 |
| | $\alpha_2$, $\beta_1$, $\beta_2$, $\kappa$ | **34360**‡ | 33182 | **34122**‡ | 33248 |
| | $\alpha_2$, $\beta_1$, $\beta_2$, $\kappa_1$,$\kappa_2$ | 34505 | **33071**‡ | 34143 | **32837**‡ |
| $Forward_2$ | | | | | |
| | $\alpha_2$, $\beta$ | 35304 | 34831 | 35279 | 34872 |
| | $\alpha_2$, $\beta_1$, $\beta_2$ | 34642 | 33732 | 34556 | 33758 |
| | $\alpha_2$, $\beta$, $\kappa_1$ | 34743 | 34046 | 34610 | 33959 |
| | $\alpha_2$, $\beta$, $\kappa_2$ | 35432 | 34579 | 35274 | 34612 |
| | $\alpha_2$, $\beta$, $\kappa$ | 34824 | 33641 | 34702 | 33619 |
| | $\alpha_2$, $\beta$, $\kappa_1$,$\kappa_2$ | 34862 | 33761 | 34594 | 33661 |
| | $\alpha_2$, $\beta_1$, $\beta_2$, $\kappa_1$ | 34430 | 33462 | 34181 | 33359 |
| | $\alpha_2$, $\beta_1$, $\beta_2$, $\kappa_2$ | 34727 | 33366 | 34508 | 33324 |
| | $\alpha_2$, $\beta_1$, $\beta_2$, $\kappa$ | 34372 | 33205 | 34129 | 33256 |
| | $\alpha_2$, $\beta_1$, $\beta_2$, $\kappa_1$,$\kappa_2$ | 34517 | 33095 | 34152 | 32851 |
| $Forward_3$ | | | | | |
| | $\alpha_2$, $\beta$, $\zeta$ | 35484 | 34993 | 35297 | 34883 |
| | $\alpha_2$, $\beta_1$, $\beta_2$, $\zeta$ | 34797 | 33841 | 34570 | 33748 |
| | $\alpha_2$, $\beta$, $\kappa_1$, $\zeta$ | 34923 | 34206 | 34630 | 33965 |
| | $\alpha_2$, $\beta$, $\kappa_2$, $\zeta$ | 35611 | 34742 | 35288 | 34633 |
| | $\alpha_2$, $\beta$, $\kappa$, $\zeta$ | 35004 | 33803 | 34717 | 33629 |
| | $\alpha_2$, $\beta$, $\kappa_1$, $\kappa_2$, $\zeta$ | 35042 | 33922 | 34624 | 33656 |
| | $\alpha_2$, $\beta_1$, $\beta_2$, $\kappa_1$, $\zeta$ | 34590 | 33579 | 34192 | 33346 |
| | $\alpha_2$, $\beta_1$, $\beta_2$, $\kappa_2$, $\zeta$ | 34882 | 33475 | 34516 | 33313 |
| | $\alpha_2$, $\beta_1$, $\beta_2$, $\kappa$, $\zeta$ | 34533 | 33335 | 34140 | 33255 |
| | $\alpha_2$, $\beta_1$, $\beta_2$, $\kappa_1$, $\kappa_2$, $\zeta$ | 35229 | 34091 | 34145 | 32841 |

*See *Materials and methods* for main differences between each of the three model-sensitive (MS) models used.

†Abbreviations: learning rate for second-stage ($\alpha_2$); inverse temperature for first-stage ($\beta_1$) and second-stage ($\beta_2$); $\beta$ is when $\beta_1 = \beta_2$; perseveration for first-stage ($\kappa_1$) and second-stage ($\kappa_2$); $\kappa$ is when $\kappa_1 = \kappa_2$; eligibility trace ($\lambda$); $\zeta$ is a weight given to state-transition model testing.

‡Best fitting MS model variant for the respective subject and analysis type.
