# Supplementary material for: Combined model-free and model-sensitive reinforcement learning in non-human primates
Source: PLoS Comput Biol. 2020 Jun 22;16(6):e1007944. doi: 10.1371/journal.pcbi.1007944 (PMC7332075; doi:10.1371/journal.pcbi.1007944)
Supplement: S5 Table — (PDF) [file pcbi.1007944.s014.pdf]

Parameters*	Fixed-effects BIC sum		Mixed-effects BIC_{int}	
	C	J	C	J
α, β, β, ω	35306	34893	35148	34834
α, β, κ_1, ω	34807	34144	34522	33930
α, β, κ_2, ω	35435	34642	35148	34592
α, β, κ, ω	34880	33742	34616	33600
α, β, λ, ω	35451	34900	35171	34775
α_1, α_2, β, ω	35464	35028	35168	34814
α, β_1, β_2, ω	34515	33638	34246	33548
α_1, α_2, β_1, β_2, ω	34577	33735	34313	33585
α_1, α_2, β, κ_1, ω	34973	34292	34561	33923
α_1, α_2, β, κ_2, ω	35593	34779	35195	34582
α_1, α_2, β, κ, ω	35045	33891	34644	33617
α_1, α_2, β, λ, ω	35602	35023	35191	34733
α, β_1, β_2, κ_1, ω	34380	33432	33948	33215
α, β_1, β_2, κ_2, ω	34602	33269	34194	33217
α, β_1, β_2, κ, ω	**34326**†	33199	**33898**†	33189
α, β_1, β_2, λ, ω	34652	33640	34244	33499
α, β, κ_1, κ_2, ω	34928	33861	34508	33673
α, β, κ_1, λ, ω	34966	34216	34542	33913
α, β, κ_2, λ, ω	35580	34650	35167	34542
α, β, κ, λ, ω	35036	33813	34640	33566
α_1, α_2, β_1, β_2, κ_1, ω	34468	33553	33986	33252
α_1, α_2, β_1, β_2, κ_2, ω	34663	33367	34265	33167
α_1, α_2, β_1, β_2, κ, ω	34422	33346	33948	33239
α_1, α_2, β_1, β_2, λ, ω	34709	33748	34302	33541
α, β_1, β_2, κ_1, κ_2, ω	34468	**33063**†	33904	**32807**†
α, β_1, β_2, κ_1, λ, ω	34528	33473	33952	33182
α, β_1, β_2, κ_2, λ, ω	34739	33272	34202	33075
α, β_1, β_2, κ, λ, ω	34475	33249	33906	33197
α_1, α_2, β, κ_1, κ_2, ω	35095	34010	34558	33642
α_1, α_2, β, κ_1, λ, ω	35125	34355	34576	33889
α_1, α_2, β, κ_2, λ, ω	35731	34769	35200	34552
α_1, α_2, β, κ, λ, ω	35194	33951	34668	33566
α, β, κ_1, κ_2, λ, ω	35087	33935	34524	33612
α_1, α_2, β_1, β_2, κ_1, κ_2, ω	34556	33186	33950	32843
α_1, α_2, β_1, β_2, κ_1, λ, ω	34612	33599	33986	33234
α_1, α_2, β_1, β_2, κ_2, λ, ω	34795	33380	34255	33180
α_1, α_2, β_1, β_2, κ, λ, ω	34568	33392	33940	33235
α, β_1, β_2, κ_1, κ_2, λ, ω	34616	33105	33903	32917
α_1, α_2, β, κ_1, κ_2, λ, ω	35246	34071	34563	33606
α_1, α_2, β_1, β_2, κ_1, κ_2, λ, ω	34699	33231	33931	32821

*All $Hybrid$ model variants tested used $SARSA$ MF model and the $Forward_1$ MS model (see full text for details). Abbreviations: learning rate for first-stage (α_1) and second-stage (α_2); α is when $\alpha_1 = \alpha_2$; inverse temperature for first-stage (β_1) and second-stage (β_2); β is when $\beta_1 = \beta_2$; perseveration for first-stage (κ_1) and second-stage (κ_2); κ is when $\kappa_1 = \kappa_2$; eligibility trace (λ); ω is the model-sensitive weight. †Best fitting $Hybrid$ model variant for the respective subject and analysis type.
