# Supplementary material for: Combined model-free and model-sensitive reinforcement learning in non-human primates
Source: PLoS Comput Biol. 2020 Jun 22;16(6):e1007944. doi: 10.1371/journal.pcbi.1007944 (PMC7332075; doi:10.1371/journal.pcbi.1007944)
Supplement: S6 Table — (PDF) [file pcbi.1007944.s015.pdf]

Model[‡]/Parameters[§]	Subject	Fixed-effects[*] BIC sum	% BIC $Hybrid$ better	% LRT $Hybrid$ better	Mixed-effects[†] BIC_{int}	PEP vs. $Hybrid$	Predictive choice probability
$SARSA$ model							
α_1, α_2, β_1, β_2, κ_1, κ_2, λ	C	35697	100	100	35260	< 0.001	0.569
α, β, κ, λ	J	33931	93	100	33750	< 0.001	0.563
$Forward_1$ model							
α_2, β_1, β_2, κ	C	34360	43	70	34122	< 0.001	0.579
α_2, β_1, β_2, κ	J	33182	63	93	33248	< 0.001	0.568
$Hybrid$ model							
α, β_1, β_2, κ, ω	C	34326	-	-	33898	-	0.581
α, β_1, β_2, κ_1, κ_2, ω	J	33063	-	-	32807	-	0.572
**$Hybrid+$ model**							
**α, β_1, β_2, κ, L_1, L_2, L_3, ω**	**C**	**33247**	**7**	**7**	**32441**	**> 0.999**	**0.595**
**α, β_1, β_2, κ_1, κ_2, L_1, L_2, L_3, ω**	**J**	**28888**	**0**	**0**	**28659**	**> 0.999**	**0.614**

[*] BIC, Bayesian Information Criterion (lower values correspond to better models) sum and % of sessions where the $Hybrid$ model was better; % of sessions with LRT, likelihood-ratio test favouring the $Hybrid$ model.

[†] BIC_{int}, is the integrated BIC (see text); PEP, is the Bayesian exceedance probability (Rigoux et al. 2014) measuring the likelihood that each model is the most common when tested against the $Hybrid$ model.

[‡]Both $Hybrid$ and $Hybrid+$ (in bold as it was the overall best model) models included the $SARSA$ model (as model-free) and the $Forward_1$ (as model-sensitive).

[§] Abbreviations: learning rate for first-stage (α_1) and second-stage (α_2); α is when $\alpha_1 = \alpha_2$; inverse temperature for first-stage (β_1) and second-stage (β_2); β is when $\beta_1 = \beta_2$; perseveration for first-stage (κ_1) and second-stage (κ_2); κ is when $\kappa_1 = \kappa_2$; eligibility trace (λ); $L1_1$, $L2_2$ and L_3 are the reinforcement strength (or aversion) for high, medium and low reward, respectively (see text for full details); ω is the model-sensitive weight.
