# Supplementary material for: Combined model-free and model-sensitive reinforcement learning in non-human primates
Source: PLoS Comput Biol. 2020 Jun 22;16(6):e1007944. doi: 10.1371/journal.pcbi.1007944 (PMC7332075; doi:10.1371/journal.pcbi.1007944)
Supplement: S7 Table — (PDF) [file pcbi.1007944.s016.pdf]

Predictors‡	Fixed-effects*		Mixed-effects†	
	C	J	C	J
Const	0.02 (< 0.01)§	0.02 (0.01)§	0.02 (0.01)§	0.03 (0.01)§
F_t	0.14 (0.04)§	0.41 (0.07)§	0.23 (0.03)§	0.51 (0.05)§
R_{t-1}	*0.24 (0.04)*§	*-0.66 (0.05)*§	*0.29 (0.03)*§	*-0.69 (0.07)*§
T_{t-1}	0.08 (0.02)§	0.03 (0.02)	0.09 (0.02)§	0.06 (0.02)§
$R_{t-1} \times T_{t-1}$	*0.23 (0.05)*§	*0.20 (0.03)*§	*0.30 (0.04)*§	*0.22 (0.04)*§
R_{t-2}	*-0.14 (0.02)*§	*-0.09 (0.02)*§	*-0.14 (0.02)*§	*-0.04 (0.02)*§
T_{t-2}	0.03 (0.02)	-0.03 (0.02)	0.06 (0.02)§	0.08 (0.01)§
$R_{t-2} \times T_{t-2}$	*0.09 (0.04)*	*0.07 (0.03)*	*0.13 (0.03)*§	*0.10 (0.03)*§
R_{t-3}	*-0.07 (0.02)*§	*-0.02 (0.02)*	*-0.08 (0.02)*§	*-0.03 (0.02)*§
T_{t-3}	0.01 (0.02)	-0.01 (0.02)	0.05 (0.04)§	0.09 (0.02)§
$R_{t-3} \times T_{t-3}$	*0.02 (0.04)*	*-0.01 (0.03)*	*0.14 (0.03)*§	*0.10 (0.03)*§
R_{t-4}	*-0.01 (0.02)*	*0.04 (0.02)*	*-0.07 (0.02)*§	*-0.04 (0.02)*§
T_{t-4}	0.01 (0.02)	0.01 (0.02)	0.06 (0.02)§	0.03 (0.01)§
$R_{t-4} \times T_{t-4}$	*-0.02 (0.04)*	*0.04 (0.02)*	*0.10 (0.03)*§	*0.07 (0.02)*§
R_{t-5}	*-0.03 (0.02)*	*0.04 (0.02)*¶	*0.11 (0.04)*§	*-0.04 (0.01)*§
T_{t-5}	< 0.01 (0.02)	0.02 (0.02)	0.06 (0.03)	0.02 (0.02)
$R_{t-5} \times T_{t-5}$	*0.06 (0.04)*	*0.04 (0.05)*§	*0.14 (0.03)*§	*0.16 (0.04)*

*Values of fixed-effects results are mean (SEM) of the regression coefficients across sessions.

†Values of mixed-effects results are the regression coefficients (SE).

‡For the given trial t, the variables used were: F was used to model (linearly-increasing) fatigue by counting the trials in the session; C is first-stage choice (1=car picture, 0=watering can picture); R is outcome level (assumed as continuous and with low=1, medium=2, high=3); and T is transition (rare=1, common=0). Const is the constant term. Predictors were mean centred and continuous variables were also scaled by dividing them by two SD (adjustments made before the computation of the interaction terms). In italic are the predictors of interest.

§Significance at the 0.01 level. ¶Significance at the 0.05 level.
