# Supplementary material for: Combined model-free and model-sensitive reinforcement learning in non-human primates
Source: PLoS Comput Biol. 2020 Jun 22;16(6):e1007944. doi: 10.1371/journal.pcbi.1007944 (PMC7332075; doi:10.1371/journal.pcbi.1007944)
Supplement: Attachment — Submitted filename: Reply_to_reviewers_VeryFinalVersion.docx [file pcbi.1007944.s019.docx]

Dear Editor,

Thank you for considering the manuscript for publication.

We have now revised the initially submitted manuscript, taking into account the comments from the reviewers.

We would like to sincerely thank the reviewers for their exceptionally meticulous revisions from which our newly submitted manuscript has greatly benefitted.

Please find below our replies to each Reviewers’ comments. For convenience, the Reviewers’ text will be shown in black, our responses will be shown in red, and changes to the manuscript highlighted in blue.

Bruno Miranda

(on behalf of all authors)

**Reviewer #1:**
The authors describe a study in which they collected an extensive dataset on the two-step task in two non-human primates. The large dataset allowed them to carry out a very detailed modeling analysis. They found that the animals had strong model-sensitive components to their learning behavior. They also compared the results of RL models to a logistic regression analysis and showed that although the models were in agreement, the RL model provided more detailed insight into the behavior.

This is a nice paper on an important topic. Although the two-step task has been extensively used in human studies, the human work has generally relied on datasets with relatively small numbers of trials per subject, which makes detailed behavioral modeling difficult. In addition, little is yet known about the neural mechanisms underlying the behaviors captured by the two-step task, and implementation in a macaque model opens the door to future studies.

I have only two comments. The paper was clear, straightforward and detailed.

Comments
1. I liked the way the authors set out terminology in the introduction, making a distinction between “model-sensitive” and model-based approaches. I think this could be taken a bit farther. Or, at least, what I would recommend is that a bit more detail could be given in the discussion, to suggest that a complete model-based approach to this task would include explicit knowledge of transition probabilities, and the non-stationary nature of the reward outcomes. Even the models which have been put forward as “model-based” for this task have been mostly model-free, with only knowledge of the transition probabilities added. Related to this, I think the highly over-trained approach used in this study makes it more likely that a true model-based or model-sensitive approach would be developed by the animals. This is not often the case in human studies, where the participants are likely still learning the transition probabilities, or at least it is not clear if the participants have completely learned the transition probabilities.

Thank you for this comment.

We have added material to both the introduction and the discussion to comment on the distinction between what we call pure model-based (MB) approaches (using prospective planning) and model-sensitivity. The Akam et al 2015 study shows that we cannot conclude from apparently MB behaviour that choices were made with a particular MB process model – and, indeed, we use the Hybrid+ model-sensitive (MS) perseveration to argue exactly for this. For instance, in the discussion of MS perseveration, we say (new text in blue):

“In particular, an excessive influence of the immediately previous trial motivated the novel Hybrid+ model, which closely reproduced the observed choice behaviour. Its extra parameters changed the influence of the action chosen on that trial as a function of the reward and the transition. We considered this to be a form of sophisticated, MS perseveration, dependent on a one-step working-memory-for-state representation (Akam, Costa and Dayan, 2015). Given the precise structure of our version of the task, this sort of MS perseveration could have underpinned apparently ideal MS behaviour, adjusting to the changing second-state contingencies as soon as they were discovered. This underscores the observation that many potential process models could lead to appropriate choices, even without the sort of explicit forward planning that is the paradigmatic signature of model-based planning. Indeed it has been argued that the use of one-step working-memory-for-state representations such as this by a MF system is a possible implementation of apparently MB planning when learning suffices to generate such representations (Akam, Costa and Dayan, 2015), and it is interesting to speculate that the subjects might have adjusted their behaviour even more optimally using MS perseveration had training continued further.

However, this MS perseveration did not persist beyond a single trial. Thus, and most critically, model comparison showed that this effect co-existed with conventional MS and MF reasoning.”

We tested explicitly MS accounts that learned transition probabilities (*Forward_3_*) against those that had their true values ‘baked in’ (*Forward_1_*), presumably from the extensive prior experience. The latter accounts fit better.

---

2. Although beyond the scope of the current paper, it would be interesting to look at acquisition of model-sensitive behavior while the task was being learned. I see that the authors have done some analyses over the sessions reported in the paper, but presumably this followed previous extensive experience on the task. I think this would be an interesting area for future work. Do naïve animals look truly model-free?

Thank you for this comment.

Unfortunately, as the task underwent many changes throughout the training process (e.g., changes in reward/delay amounts, use of different stimuli, changes to how frequently outcome levels were updated. etc.), it is not possible to directly compare these early training results with the data from the final task variant. However, we would like to share two independent findings we observed from the early training stages.

First, in initial phases of learning the task the outcome levels only differed in the magnitude of the rewards. The training proceeded well, with significant differences emerging gradually in first-stage choice behaviour between common and rare trials for the highest outcome level (i.e., repeat if common-high, switch if rare-high). This was a signature of MS behaviour. However, the expected difference between transition types for the lowest outcome level was hard to elicit (i.e., switch common-low was similar to switch rare-low), which reflected more of a MF strategy. In short, it appeared as though MS behaviour for large positive outcomes occurred faster than MS behaviour for small (or non-rewarded) outcomes. Two possible reasons were considered: i) the overall subjective value of the low outcome level was not low enough, and ii) the subjective value of the different outcome levels was not different enough to promote further optimisation of the task. We therefore tried negative outcomes (by delivering a diluted quinine solution) for the low outcome level. This rapidly had the consequence of evoking a repeat on rare-low trials (i.e., a MS signature), but also led to reduced task motivation. We thus discontinued quinine and instead introduced delays in addition to increasing the difference in the reward amounts per outcome level. We also introduced three (rather than two), possible motor responses to discourage motor biases. This was very successful in achieving the results presented in this manuscript. Hence, the addition of delays was used merely to make each outcome level more salient, rather than to examine differences in rewards and delays in MS/MF behaviour, a point raised by Reviewer 2.

Second, in another experimental phase of the training protocol, the stimuli used as well as the background colours for each state varied every day but keeping the 70%/30% state-transition structure constant throughout the session. This allowed us to examine new transition matrix learning on every session. As a whole, the results were consistent with the “typical MF-RL” t-1 choice profile (i.e., higher probability of repeating the same first-stage choice as a function of increasing reward, regardless of transition type; i.e., a main effect of reward only) in these earlier phases of learning. As this did not easily generate our desired MS behaviour (i.e. reward x transition interaction), we did not perform extensive testing with this task variant. Nonetheless, this anecdotal evidence is not consistent with the idea that goal-directed or MB learning is prominent in initial phases of learning (Dickinson and Balleine, 2002), given the higher uncertainty of MF-RL at initial stages, as suggested by some theoretical views (Daw et al., 2005). In fact, there is a contemporary debate about what “should” happen in these initial phases of learning: some model-free meta-control?; just some generically useful behavioural routines that allow MB behaviour to “get off the ground”; or episodic RL – local repetitions of apparently good choices. This topic deserves further exploration in future experimental work.

We have commented on these points in the revised Discussion (new text in blue):

“In keeping with the observation that many manipulations reduce MS control without increasing MF control, it has been suggested that MF influences might instead arise from MS reasoning with incorrect or incompetent models (da Silva and Hare, 2019). The MS perseveration effect could perhaps be seen as an example of this (albeit one that should have been exploited more rather than less). There is, of course, a large range of possible flawed models; however, the extensive training (and the large value of ω we found) perhaps suggest that this problem might be less severe in our study, perhaps because of the extensive training that our subjects enjoyed. This characteristic of the study made it impossible for us to examine the progressive acquisition of the model – this is an important task for the future. Note also the differences between our design and that of the original human studies (Daw et al. 2011). Of greatest theoretical import were the adjustments to the second stage reward structure that were designed to make MS choice more lucrative for the subjects (Kool et al. 2016) – this underpins the potential benefit of MS perseveration. The addition of delays to reward (in addition to different reward magnitudes) was intended to increase the salience of the differing outcome levels; it had no effect on the informational structure of the task.”

**Reviewer #2:**
In this work by Miranda et al, authors have studied performance of nonhuman primates in the Daw’s 2-step decision task that is typically used to dissociate a model-based planning strategy from model-free habitual response pattern. This is an important study that advances our knowledge about decision making in nonhuman primates as well as about the 2-step task that currently serves as an important behavioral paradigm in decision neuroscience. Therefore, I believe that this work is suitable for publication in PLoS CB. I have a couple of suggestions that authors might want to consider:

1) The main issue with the manuscript, as far as I understand, is that the difference between model-based (MB) and what authors called model-sensitive (MS) is not clear and a bit misleading. The MS is related to the nice computational paper of Akam et al (2015, PCB). The issue is that without full knowledge of Akam et al.’s paper, readers will not understand the difference between MB and MS, and most likely interpret the MS as a variant of MB strategy. In fact, however, MS seems to be a variant of MF that does not include prospective planning (given that the Hybrid+ model explains data better than Hybrid, and as noted by authors in page 15, this model is although MB-like, but in fact is a variant of MF). The MS model is therefore a model of “sophisticated habit” (Akam et al, 2015) rather than planning.

I think the paper will be improved if it is explicitly said in the introduction that MS is a variant of MF. Related to this point, I also think that both the title and abstract suggest that MS is a variant of MB, particularly given the literature in human version of this task about coexistence of MB and MF, and therefore it is better to revise them to avoid misinterpretation.

Thank you for this comment.

We apologise for being insufficiently clear on this important point. The Hybrid model is exactly an instance of the combination of (conventional) MB and MF reasoning that previous studies on humans have assumed. That is, the ‘forward’ model that is included in the Hybrid model involves the prospective calculation that is inherent to MB reasoning. However, we call this component MS rather than MB to acknowledge Akam et al.’s insight that, in versions of the task such as ours (and the previous human studies), it is possible to generate the same answer as prospective planning, but without engaging explicitly in this implied MB process. Thus, we consider MS more of a variant of MB than MF, and hence this is reflected in the title and abstract accordingly. Nonetheless, this model mimicry is hard to resolve without neural data.

This conclusion is reinforced by the MS perseveration that is inherent to Hybrid+. In fact, this dependence could have been used to generate behaviour that would have been considered perfect from a prospective viewpoint, given the particular second-stage outcome statistics that we employed. In the previous version, we suggested that this MS perseveration might have operated through manipulating MF values. However, in retrospect, we realized that this is confusing – since we do not have any way of knowing that this is true – thus, we now confine ourselves to commenting on the behavioural dependencies.

In the “Results” section, we now say:

“Given the structure of the environment, with non-overlapping second-stage reward statistics, it would have behoved the subjects to have relied just on this sort of MS perseveration rather than the progressive learning of second-stage values inherent to both the MF and MS methods in the Hybrid+ model. However, this does not describe their behaviour accurately.”

and in the “Discussion” section we now say (new text in blue):

“In particular, an excessive influence of the immediately previous trial motivated the novel Hybrid+ model, which closely reproduced the observed choice behaviour. Its extra parameters changed the influence of the action chosen on that trial as a function of the reward and the transition. We considered this to be a form of sophisticated, MS perseveration, dependent on a one-step working-memory-for-state representation (Akam, Costa and Dayan, 2015). Given the precise structure of our version of the task, this sort of MS perseveration could have underpinned apparently ideal MS behaviour, adjusting to the changing second-state contingencies as soon as they were discovered. This underscores the observation that many potential process models could lead to appropriate choices, even without the sort of explicit forward planning that is the paradigmatic signature of model-based planning. Indeed it has been argued that the use of one-step working-memory-for-state representations such as this by a MF system is a possible implementation of apparently MB planning when learning suffices to generate such representations (Akam, Costa and Dayan, 2015), and it is interesting to speculate that the subjects might have adjusted their behaviour even more optimally using MS perseveration had training continued further.

However, this MS perseveration did not persist beyond a single trial. Thus, and most critically, model comparison showed that this effect co-existed with conventional MS and MF reasoning.”

---

2) Monkeys have been overtrained in this task, and therefore, it is not surprising that they follow a MF strategy. I suggest to report an additional analysis to test whether there is a difference between the first and the last session of training for each monkey, by fitting models (including Hybrid and Hybrid+) to the first and last session separately and test whether and if model evidence or likelihood changes between these two sessions.

Thank you for this comment.

First, it is important to note that the first session shown in the paper is not the first session since there was a training path for both subjects. As raised in response to Reviewer 1, Point 2, we cannot make direct comparisons between the current data and the data in prior testing sessions because of task adjustments (stimuli used, reward amounts, etc). In addition, in very early training stages when the subjects were first exposed to new stimuli or new transition probabilities, their behaviour had a tendency to follow a response profile more in line with a MF learner – i.e., only a main effect of reward (e.g., switch common-low was similar to switch rare-low). As training continued with the same stimuli and transition probabilities, our measures of MS-weight (not MF-weight) increased. This is, in a way, consistent with some arguments from Dickinson and Balleine (Dickinson and Balleine, 1995, 2002) that rodents do not habitize more complicated tasks (e.g., two action, two outcomes) even with extensive training - albeit not as extensive as ours. Once the subjects exhibited stable behaviour (which was MS), we started electrophysiological recordings. The data presented here is the full behavioural dataset obtained during electrophysiological recordings.

Secondly, we would like to clarify that our presented evidence does not suggest that overtraining made monkeys more likely to follow a MF strategy. As stated in our “Results” section “We found no significant relationship between session number and the main effect of previous reward, nor a reduction in the ω parameter across sessions (S6 FigA-B). On the other hand, the previous reward × transition interaction effect reduced across sessions in both subjects (S6 FigC).”. To note that more negative previous reward x transition interaction effect is supportive of a stronger MS-RL strategy, so there is some evidence that this MS-effect diminished slightly with overtraining, though the weighting parameter (ω) value near 90% clearly shows the MS component dominated.

Additionally, we provide other reasons for why our findings are more in line with the observed MS dominance, as stated in our “Discussion” section: “That MS control dominated more here (ω near 90%) than in recent human studies (ω approximately 40-60%; [8,36]; or ω → 0; [24]) could result from the non-stationarity of the outcomes (changing every 5-9 trials), which should optimally favour the more flexible, MS, controller (Daw, Niv and Dayan, 2005) or be another effect of the extensive training, as also seen in human studies (Economides et al., 2015). It could arise from an increase in the efficiency of the implementation of MS reasoning, for instance from reducing its computational cost and increasing its speed.”

Nonetheless, we appreciate the Reviewer’s suggestion, and have conducted additional analyses to assess whether there were changes in MF/MS-weights across time. We examined the difference in model evidence and likelihood changes between the first and last session for each subject when we fitted separately (i.e. fixed-effects modelling) the models (including Hybrid and Hybrid+).

Results of the first and last session for Subjects C/J as well as their respective measures of model fitting (best fit shown in **BOLD**):

**Comparison between first and last session for Subject C**

| Model |  | BIC | NegLL/choice |
| --- | --- | --- | --- |
| **SARSA** |  |  |  |
|  | First session | 1398.8 | -1.0966 |
|  | **Last session** | **1019.2** | **-1.1461** |
| **FORWARD1** |  |  |  |
|  | First session | 1366.7 | -1.0711 |
|  | **Last session** | **992.7** | **-1.1157** |
| **HYBRID** |  |  |  |
| (MS weight = 0.83) | First session | 1365.4 | -1.0649 |
| (MS weight = 0.88) | **Last session** | **996.5** | **-1.1131** |
| **HYBRID+** |  |  |  |
| (MS weight = 0.85) | First session | 1329.4 | -1.0206 |
| (MS weight = 0.85) | **Last session** | **980.9** | **-1.0742** |

**Comparison between first and last session for Subject J**

| Model |  | BIC | NegLL/choice |
| --- | --- | --- | --- |
| **SARSA** |  |  |  |
|  | First session | 1557.5 | -1.1800 |
|  | **Last session** | **1321.3** | **-1.1825** |
| **FORWARD1** |  |  |  |
|  | First session | 1501.5 | -1.1317 |
|  | **Last session** | **1276.1** | **-1.1356** |
| **HYBRID** |  |  |  |
| (MS weight = 0.86) | First session | 1498.2 | -1.1216 |
| (MS weight = 0.93) | **Last session** | **1281.3** | **-1.1345** |
| **HYBRID+** |  |  |  |
| (MS weight = 0.88) | First session | 1272.9 | -0.9357 |
| (MS weight = 0.93) | **Last session** | **1223.2** | **-1.0642** |

And if we extend the analysis to the first 3 sessions vs. last 3 sessions (values shown are means):

**Comparison between first 3 sessions and last 3 session for Subject C**

| Model |  | Mean BIC | Mean NegLL/choice |
| --- | --- | --- | --- |
| **SARSA** |  |  |  |
|  | First session | 1293.2 | -1.0830 |
|  | **Last session** | **1169.7** | **-1.1402** |
| **FORWARD1** |  |  |  |
|  | First session | 1270.8 | -1.0638 |
|  | **Last session** | **1134.0** | **-1.1048** |
| **HYBRID** |  |  |  |
| (MS weight = 0.77) | First session | 1259.0 | -1.0483 |
| (MS weight = 0.86) | **Last session** | **1134.2** | **-1.0988** |
| **HYBRID+** |  |  |  |
| (MS weight = 0.85) | First session | 1229.8 | -1.0070 |
| (MS weight = 0.89) | **Last session** | **1115.0** | **-1.0612** |

**Comparison between first 3 sessions and last 3 session for Subject J**

| Model |  | Mean BIC | Mean NegLL/choice |
| --- | --- | --- | --- |
| **SARSA** |  |  |  |
|  | First session | 1430.7 | -1.1537 |
|  | **Last session** | **1129.1** | **-1.1741** |
| **FORWARD1** |  |  |  |
|  | First session | 1366.9 | -1.0959 |
|  | **Last session** | **1103.0** | **-1.1398** |
| **HYBRID** |  |  |  |
| (MS weight = 0.90) | First session | 1365.1 | -1.0892 |
| (MS weight = 0.87) | **Last session** | **1105.2** | **-1.1358** |
| **HYBRID+** |  |  |  |
| (MS weight = 0.92) | First session | 1183.4 | -0.9243 |
| (MS weight = 0.91) | **Last session** | **1038.9** | **-1.0456** |

This analysis revealed that the model fit improved (lower BIC scores) for both subjects from first to last session across all models. As expected, the Hybrid+ model better fit the later sessions compared to the earlier sessions, implying greater MS contribution as further training was obtained across sessions.

Thus, overall the MS-weight remained very stable across sessions, and we did not find an increase in MF-weight across sessions. Although it would be interesting to examine how the MF/MS weighting changes during learning (of transition probabilities, new stimuli, different rewards etc), this was not the aim of the current study. Moreover, due to task changes during training (different stimuli, reward magnitudes), it is not possible to directly compare the early training data to the current data (see Reviewer 1, Point 2).

We have now made these points clearer in the Discussion” (new text in blue):

“In keeping with the observation that many manipulations reduce MS control without increasing MF control, it has been suggested that MF influences might instead arise from MS reasoning with incorrect or incompetent models (da Silva and Hare, 2019). The MS perseveration effect could perhaps be seen as an example of this (albeit one that should have been exploited more rather than less). There is, of course, a large range of possible flawed models; however, the extensive training (and the large value of ω we found) perhaps suggest that this problem might be less severe in our study, perhaps because of the extensive training that our subjects enjoyed. This characteristic of the study made it impossible for us to examine the progressive acquisition of the model – this is an important task for the future. Note also the differences between our design and that of the original human studies (Daw et al. 2011). Of greatest theoretical import were the adjustments to the second stage reward structure that were designed to make MS choice more lucrative for the subjects (Kool et al. 2016) – this underpins the potential benefit of MS perseveration. The addition of delays to reward (in addition to different reward magnitudes) was intended to increase the salience of the differing outcome levels; it had no effect on the informational structure of the task.”

---

3) Please report protected exceedance probability (Rigoux et al., 2013, Neuroimage) instead of exceedance probability (for example in Table S6). The latter is shown to be too liberal (Rigoux et al., 2013, Neuroimage).

Thank you for this comment. We have made the suggested correction on Table S6 and text after computing the protected exceedance probability. The reference was also changed accordingly to Rigoux et al. 2013, Neuroimage.

**Reviewer #3:**
 In the manuscript “Combined model-free and model-sensitive reinforcement learning in non-human primates”, the authors present a detailed analysis of choice modelling on a sequential decision task in primates, adapted from the original two-step decision task in humans (Daw et al.). They use a combination of regression models and trial-by-trial RL model fitting to probe the contributions of so-called model-free and model-sensitive learning and decision components on the observed modelling. Overall, this is a very thorough and thoughtful study, contributing to the large literature on sequential decisions in this two-stage setting with probabilistic transitions, with findings that are largely confirmatory with previous literature, though extending them into non-human primate modelling. However, there are some inconsistencies and omissions in the descriptions of the models and methods that are puzzling and should be rectified, as well as a lack of clarity about the logic of the model – Hybrid+ -- that they propose as the best fit to the modelling of their two subjects.

Major concerns:
- The logic of the Hybrid+ model is somewhat confusing embedded within the relatively straightforward RL models used in the paper. It seems that the Hybrid+ model is essentially a complex form of perseveration, but it only goes one trial back and is an explicit factor that is independent for each possible type of previous trial transition x reward. I would like the authors to go further in interpreting the success of including this t-1 structure within the incremental learning RL formalism that they are using overall. It is not clear whether the authors think this statistical piece-wise influence of the previous trial is actually a learning effect, a memory effect or they are solely trying to capture the pattern in the data without probing where it enters their models as a process. Essentially, is this hybrid+ model equivalent to other forms of the hybrid model that have more interpretable parameters? – for example with a mixing parameter that depends on the previous trial? Or MS component with learning (instead of just choice) that is different for the different types of outcome experience? Do the authors think these effects are strictly one trial back or is this a useful approximation? The possible interpretations of the L_x model parameters should be discussed in more detail.

Thank you for this comment.

The simplest answer is that, given suitable strength, the MS perseveration inherent to Hybrid+ would be the perfect sort of “model-basedness” for this particular version of the task in which the second-stage outcomes are non-overlapping (and so perfectly informative). By comparison, the ‘forward’ model in Hybrid assumes progressive learning of the second stage outcome value, which is inappropriate for the sudden switches that we employed.

However, the subjects did not rely solely on this control mechanism. Instead, it seems only to have had an influence that extended one trial in the future – and incorporating it allowed the model to fit the behaviour far more proficiently, whilst leaving the ‘main’ MF and MS components of Hybrid mostly unaffected.

There was no variation left in the data that merited extending this one-trial effect more trials into the past. It is intriguing to wonder whether the subjects would progressively rely persistently on this component, as would have been ideal.

To make our points clearer about the L_x model parameters, in particular our view that this could be a memory-related process – or more specifically a “working-memory-for-state representations”; and that there is no interference (“mixing parameter”) between this new model parameters and our learning parameters, our new “Discussion” section includes the following (new text in blue):

“We considered this to be a form of sophisticated, MS perseveration, dependent on a one-step working-memory-for-state representation (Akam, Costa and Dayan, 2015). Given the precise structure of our version of the task, this sort of MS perseveration could have underpinned apparently ideal MS behaviour, adjusting to the changing second-state contingencies as soon as they were discovered. This underscores the observation that many potential process models could lead to appropriate choices, even without the sort of explicit forward planning that is the paradigmatic signature of model-based planning. Indeed it has been argued that the use of one-step working-memory-for-state representations such as this by a MF system is a possible implementation of apparently MB planning when learning suffices to generate such representations (Akam, Costa and Dayan, 2015), and it is interesting to speculate that the subjects might have adjusted their behaviour even more optimally using MS perseveration had training continued further.

However, this MS perseveration did not persist beyond a single trial. Thus, and most critically, model comparison showed that this effect co-existed with conventional MS and MF reasoning. MS perseveration could also be seen as a short-term form of sophisticated counterfactual or regret/rejoice-based influence on the unchosen action (Lohrens et al., 2007]. Indeed, other limited MS strategies have been noted in the case of serial reversals (Doll, et al., 2012).”

---

- The authors should discuss and test more fully how this version of the two-step task, with these reasonable changes in outcomes for non-human participants, potentially change the task in ways that are distinct from what is seen in humans on more standard versions of the two-step task (in which outcomes are strictly positive, though variants exist). For example, did the authors test whether the trial-1 perseveration in the Hybrid+ model (and indeed other parameters in the other models) is actually related to trials with/without delays as opposed to levels of reward outcome? Or, alternatively, is reward omission different in some way from trials that end with a reward, even if delayed. It seems that the model fitting uses the reward amount (large, small, none) and not the delay for model fitting, so this feature of the task is effectively ignored in all the models tested. Even so, from the parameter fits for L_x it seems these other factors are reasonable possibilities, rather than parametric outcomes as assumed in the model fitting performed through the study. This is a distinct qualitative alternative interpretation for the factors influencing learning and decision making on this task, so should definitely be discussed.

Thank you for this comment.

We apologize for any confusion regarding the use of delays in our task. We included delays in addition to differing reward magnitudes to increase the salience of the second-stage outcomes for the subjects (see also reply to reviewer 1, Point 2), not to examine the independent influence of reward and delay in MF/MS behaviour. The precise reward amount for the “high” and “medium” outcomes (note “low” outcome had no reward) varied slightly across trials but with non-overlapping magnitudes (e.g., for Subject C, high reward ranged on average from 682-962 ms of juice flow, and medium reward ranged on average from 117-390 ms of juice flow). This “within outcome-level reward variability” was used to promote constant valuation of the reward amount and aid the computational model fitting procedure. In contrast, there was no variability in the delays; these were fixed throughout the experiment (e.g., for Subject C: 750 ms for “medium” outcome, 2500 ms for “low” outcome (note “high” outcome had no delay)). As the reward and delay amounts were non-overlapping between outcome levels, these should not have left the subjects confused. The constant delays (i.e., lack of across trial variability) meant using delays (rather than rewards) provided a poor fit in the computational model fitting procedure and thus were not included. However, we appreciate this may not have been clear and have made changes to the manuscript accordingly. It would be interesting to look in the future at alternative designs with richer information associated with delays.

The “Methods” section now states (new text in blue):

“For the model fitting r(_2,t_) corresponded to the amount of juice delivered at trial t divided by the maximum amount of juice obtained by the subject within the entire respective session. Note that because the delay amounts were fixed for outcome level and hence contained no variability across trials, they were not explicitly included in the model-fitting.”

The “Discussion” now comments (new text in blue):

“Note also the differences between our design and that of the original human studies (Daw et al. 2011). Of greatest theoretical import were the adjustments to the second stage reward structure that were designed to make MS choice more lucrative for the subjects (Kool et al., 2016) – this underpins the potential benefit of MS perseveration. The addition of delays to reward (in addition to different reward magnitudes) was intended to increase the salience of the differing outcome levels; it had no effect on the informational structure of the task.”

---

- Unlike behavioral tasks with humans, here there are only two subjects. While some readers may understand that this is completely standard for work with non-human primates, it is a very different setting for interpreting results to generalize at a population level. The authors should discuss this in the context of the RL modelling results, as there is some variation between individuals that is hard to interpret in the context of model comparison.

We agree entirely with this point. Unfortunately, if we want to know what happens in non-human primates in the lab, financial and ethical considerations make the standard of two subjects per experiment the norm. This means that we cannot make statements that generalise to the population of all macaque monkeys, we can only make statements about the particular macaque monkeys in our lab. Nevertheless, because we have many more trials available to us than with human studies, we can make more precise statements about these monkeys than is typical in humans, and we can make these statements with confidence. These statements can be about the individual monkeys (within monkey analyses), or about the pair of monkeys we are studying (Fixed or random effects analyses), but cannot generalise outside this population.

We believe that these analyses are nevertheless valuable. These types of data allow the development of rich models (as we have tried here) and allow case demonstrations (for example, we know that monkeys can exhibit model-sensitive behaviour in the lab, because we have 2 individuals that do).

It is notable that our set-up has many other types of generalisation issues that are common with most human tasks. For example, neither our nor most human tasks can generalise to different training regimes, nor different reward types, nor different instantiations of model-based behaviour. Hence we cannot make general claims about all of model-based behaviour, nor, in our case, about all macaques. We want to be clear though, that this does not mean that the claims we *are* making are less statistically robust. It just means they are limited to the situations, or animals that we have studied.

Whilst such generalisation is clearly essential when comparing different patient groups, for example, it is not clear to us that it is essential when trying to delineate the detailed patterns of behaviour. We certainly believe that both types of study (high N vs high trial-number) are valuable. We also note that we have separated all results into the two monkeys, and it was striking how similar the behavioural pattern was between the two monkeys.

We have amended the discussion to make it completely clear that we cannot generalise to the population:

“The standard for primate electrophysiology studies is to use two subjects. Our limited sample size makes it impossible to generalize our RL modelling results to the population. However, the overall behavioural pattern between our two subjects was similar, and the model comparison revealed that the main winning models shared the same principles. Moreover, by obtaining thousands of trials across dozens of sessions we were able to examine MF and MS influences on behaviour at a level of detail not achievable in previous versions of the two-step task in humans (Economides et al. 2015). Note that the small subject number does not have implications for the statistical robustness of the results. Because our inference is limited to the subject pool that we studied, and we do not try to generalise to the population, the robustness of the results is dictated by the number of trials, not the number of subjects.”

---

- The text is confusing about the actual features of the best-fitting MF model. In table 1. SARSA has best fit values for lambda (eligibility trace). But the discussion states “It was also notable that the best-fitting MF model 285 involved no eligibility trace (i.e., λ = 0); this implies, for instance, that it takes the MF 286 component at least two trials to change its estimate of the value of a first-stage action 287 following a change after the second-stage.” Also, the results suggest that the eligibility term is not included for the SARSA component of the Hybrid model, even though it appears this term is indeed present in the best fitting MF model. It should be made more explicit whether the hybrid model actually uses the best fit SARSA or not. If not allowing eligibility traces in the MF part of the hybrid model, explain the logic here. If the authors seek to keep the hybrid model with a work fitting SARSA formulation for some reason, then model comparison for the model-free part should also be done without the eligibility trace... how well can the model free part of the hybrid model do on its own?

We would like to thank the reviewer for this comment. We will start by explaining our logic in order to avoid misinterpretation of the “Results” and “Discussion” sections; and then we will explain how we tried to make this point clearer in the text.

First, when we fitted MF-RL models (SARSA, Q-learning), MS-RL models (Forward 1, 2 and 3) we allowed each combination of parameters per model. Then, model comparison analyses determined the best fit model and parameter combination for each MF and MS-RL. As correctly mentioned by the reviewer, the winning model for MF-RL was the SARSA model (not the Q-learning) that included an eligibility trace – as shown in Table S1.

For the Hybrid model, we decided to first make the choice between the best fit MF and MS-RL models (meaning choosing between SARSA vs. Q-learning – for MF-RL; and Forward 1 vs. Forward 2 vs. Forward 3 – for MS-RL) but allowing a new combination of parameters for each of these model types. As can be seen on table S5, we have allowed for the Hybrid model to have a SARSA model (in addition to a Forward1 component) with and without eligibility trace. Nonetheless, the best combination of parameters for the Hybrid model was a SARSA without eligibility-trace. And this is the Hybrid model we then refer to in the main text.

In regard to the question “how well can the MF part of the hybrid model do on its own?”, we have made that comparison and found that it was not as good as the Hybrid:

- same SARSA as the one used in the winning Hybrid (i.e. SARSA without the eligibility trace):

Subject C: Fixed BIC 35934 / Mixed BIC 35637; Subject J:Fixed BIC 34370 / Mixed BIC 34049;

vs.

- the best Hybrid:

Subject C: Fixed BIC 34326 / Mixed BIC 33898; Subject J: Fixed BIC 33063 / Mixed BIC 32807.

We appreciate that the description of this particular issue needed further refinement in the main text. Therefore, the new manuscript version includes some amendments:

In the “Results” section (new text in blue):

“This winning approach combined the SARSA MF model (a better pure MF approach than Q-learning) without an eligibility trace parameter with the Forward1 MS model (the best pure MS approach) for which the state-transition probabilities are assumed known from the beginning of the task (see Materials and methods for explanation of differences between each of the MF and MS models). Note that when only a pure MF method was allowed (rather than a Hybrid combination), the best possible was SARSA with the inclusion of an eligibility trace. However, in the Hybrid model, the effect that the eligibility trace (or other parameters) is capturing could have been more parsimoniously encompassed in other components. Therefore, in fitting the Hybrid model, we again assessed which parameters were required for the MF aspect.”

And, in the “Discussion” section (new text in blue):

“ It was also notable that the SARSA model of the best-fitting Hybrid model involved no eligibility trace (i.e., λ = 0); this implies, for instance, that it takes the MF component at least two trials to change its estimate of the value of a first-stage action following a change after the second-stage.”

---

Methodological concerns (and some minor points):

- I’m not sure it is reasonable to perform the correlation between the regression interaction effect and the model-weighting parameter in Hybrid+ across subjects (nor for the residuals). Why are these correlations computed over subjects but the one regarding perseveration is within subjects? There should be consistency here or the methodological justification should be explained.

Thank you for this comment.

We agree with the reviewer that a methodological justification is lacking for why we correlated across subjects for some parameters (ω and β) and within subjects for other parameters (κ). As a consequence, we made changes on the new version of the manuscript (see below).

We have modified the figure S5 and plotted the correlation coefficient for each subject. This is more in line with the overall “within” subjects approached used across the manuscript.

Accordingly, we have also made changes in the legend by removing the text mentioning “(separate analysis for each subject because of the different k parameters)” and “across subjects in (A and B)”, resulting in the following legend:

“S5 Fig. Correlation between logistic regression estimates and computational modelling parameters across sessions. (A) The greater the model-sensitive weight parameter ω obtained from the Hybrid+ model fitting, the more negative (i.e. the stronger the effect in the logistic regression) the regression coefficient for the reward × transition interaction. (B) Relationship between the inverse temperature parameter at first-stage choice β 1 obtained from the Hybrid+ model fitting and the residual values from the regression model (the greater the β 1 parameter, the better the logistic regression fit). (C) Positive correlation between the computational preseveration κ 1 parameter and the regression coefficient for repeat first-stage choice independently of reward and transition ~~(separate analysis for each subject because of the different κ parameters)~~. Dashed lines represent the regression line of the fit for each individual subject ~~or across subjects (in red)~~. r is the Pearson’s linear correlation coefficients and p is the p-values ~~across subjects in (A and B) and in (C)~~: top values are for subject C and bottom values are for subject J.”

In this revised figure, the correlation in S5A (ω parameter with reward x transition effect) of one of the subjects (Subject J) was not significant. Nevertheless, there was a tendency towards greater ω being associated to stronger (i.e., more negative) reward x transition effects, hence the overall pattern is similar across subjects. This finding does not change our initial claim in the “Results” section, because we did not make specific statistical statements for this particular finding:

“We found that stronger (i.e., more negative) reward × transition interaction effects were associated with greater MS ω Hybrid+ parameters (S5 FigA)”.

The reason why we performed our original analysis was because the aim of this correlation analysis was to “examine the relationship between both descriptive and computational results”. This was in a way different from the approach used for most parts of the analytical procedures across the manuscript – where we tested, for example, the best model or parameters for a particular subject. In addition, the best fitting model for Subject C had a single κ (shared for both first- and second-stage choice), whereas Subject J had two independent “κ”s for each choice (i.e., a κ1 for first-stage choice; and a κ2 for second-stage choice). Hence, an “across subjects” approach would not be ideal here – and, therefore, we decided to look at the correlation coefficients “within subjects”. Nonetheless, we appreciate this was not clearly justified and hence have reverted to all correlations being within-subject as shown in the above figure.

---

- The hybrid+ model is suggested to include a more granular perseveration effect as discussed above. But I find this model description/formulation super confusing in the methods. The methods text states L is dependent on the transition and reward on trial t (see equation subscripts), and that it is applied at choice on trial t, after the computation of the weighted hybrid MS and MF Q-values. But this is when the transition and reward on trial t is not yet known. Why is this capturing the effect of the previous trial on the present one? Is it possible that the equation is mis-written and the L factors are actually assigned based on the rewards and transition for trial t-1? I find the logic here in the formulation confusing compared to the description in the main text.

Thank you for this comment.

We believe that the confusion mentioned is generated by the notation (←) used for QHYB+ update. The value for QHYB+(t+1) is generated by the following update:

QHYB+(t) + L_i_

which is dependent on transition and reward on trial t.

To make things clearer, the formulation was amended to:

---

- BIC_int is not defined in the methods (or even mentioned in the methods as far as I can see!), despite being the criteria for mixed-effects model comparison. Should also include refs here for exceedance probability.

We apologize for this oversight.

We have now defined BIC_int in the Methods.

We have also included the required additional sections: “Model fitting procedures” as well as “Model comparison and validation procedures”; and included a reference for the (protected) exceedance probability. For convenience, the new sections are shown below:

**Have all data underlying the figures and results presented in the manuscript been provided?**
Large-scale datasets should be made available via a public repository as described in the *PLOS Computational Biology* [data availability policy](http://journals.plos.org/ploscompbiol/s/data-availability), and numerical data that underlies graphs or summary statistics should be provided in spreadsheet form as supporting information.

Reviewer #1: Yes

Reviewer #2: None

Reviewer #3: No: I do not see anywhere in the main text of the manuscript where data can be accessed.

Thank you for this comment.

We apologize for not including this information in our original submission.

Regarding “Data Sharing Methods”, we are planning to depositing all data (raw data and analysis scripts) from the “Combined model-free and model-sensitive reinforcement learning” project (i.e., behavioural and neuronal) in an appropriate public data repository (e.g. CRCNS, Dryad, G-Node or OpenNeuro) once the neuronal manuscripts are accepted (in preparation at present). However, for the present paper, we will submit the data required to replicate all study findings reported in the article as “Supporting Information files”. Therefore, we will provide two files:

>> “Miranda2020_PLOSCompBiol_RawData.mat”: a MATLAB extension file with all behavioural data (conditions, trial types, choices, reaction times and eye fixation times);

>> “Miranda2020_PLOSCompBiol_RawData_glossary.xlsx”: an Excel file where the variables present in the above .mat file are detailed for users to understand their meaning.

In the “Methods” section we now added (new text in blue):

“The data required to replicate all reported findings (together with a glossary for details) is provided as Supporting Information files.”
